# Logical Modeling and Dynamical Analysis of Cellular Networks

**DOI:** 10.3389/fgene.2016.00094

**Published:** 2016-05-31

**Authors:** Wassim Abou-Jaoudé, Pauline Traynard, Pedro T. Monteiro, Julio Saez-Rodriguez, Tomáš Helikar, Denis Thieffry, Claudine Chaouiya

**Affiliations:** ^1^Computational Systems Biology Team, Institut de Biologie de l'Ecole Normale Supérieure, CNRS UMR8197, INSERM U1024, Ecole Normale Supérieure, PSL Research UniversityParis, France; ^2^INESC-ID/Instituto Superior Técnico, University of LisbonLisbon, Portugal; ^3^Instituto Gulbenkian de CiênciaOeiras, Portugal; ^4^Faculty of Medicine, Joint Research Centre for Computational Biomedicine, RWTH Aachen UniversityAachen, Germany; ^5^Department of Biochemistry, University of Nebraska-LincolnLincoln, NE, USA

**Keywords:** regulatory and signaling networks, logical modeling, discrete dynamics, attractors, reachability analysis, simulation, T cells activation and differentiation, cell cycle control

## Abstract

The logical (or logic) formalism is increasingly used to model regulatory and signaling networks. Complementing these applications, several groups contributed various methods and tools to support the definition and analysis of logical models. After an introduction to the logical modeling framework and to several of its variants, we review here a number of recent methodological advances to ease the analysis of large and intricate networks. In particular, we survey approaches to determine model attractors and their reachability properties, to assess the dynamical impact of variations of external signals, and to consistently reduce large models. To illustrate these developments, we further consider several published logical models for two important biological processes, namely the differentiation of T helper cells and the control of mammalian cell cycle.

## 1. Introduction

As computational modeling is increasingly recognized as a necessary and valuable approach to understand dynamical features of complex biological processes, the logical framework has proved to be particularly successful to model and analyze regulatory and signaling networks (Samaga and Klamt, 2013; Albert and Thakar, 2014; Le Novère, 2015; Naldi et al., 2015). Back in 1961, following the discovery of specific gene regulation mechanisms and the delineation of the first regulatory circuits in bacteria (Jacob and Monod, 1961; Monod and Jacob, 1961), several researchers proposed to use Boolean algebra to model cellular circuits. Mitoyosi Sugita was the first to present an explicit modeling of bacterial genetic circuits with symbolic logic, applying the methods and tools of mathematics and electronics, and coining the term molecular automaton (Sugita, 1963). Soon after, Stuart Kauffman engaged in a thorough analysis of the dynamical properties of generic Boolean network models, using a synchronous update and focusing on asymptotical properties (Kauffman, 1969; Glass and Kauffman, 1973). In parallel, René Thomas rather addressed the modeling of specific regulatory circuits, in particular the network controlling lysis-lysogeny decision in bacteriophage lambda, using an asynchronous update, and progressively refining the logical formalism with the introduction of multi-valued variables, the explicit consideration of threshold values, the definition of logical parameters, etc. (Thomas, 1973; Thomas et al., 1976; Thomas, 1978). By and large, the studies of Kauffman and Thomas converged in showing that alternative stable states (or more generally alternative attractors) can be associated with different cell types, and that logical state transitions can be associated with gene expression changes over time. While Kauffman emphasized how connectivity and specific kinds of logical functions have an impact on the asymptotic network behavior, Thomas focused more specifically on the dynamical roles of simple, positive vs. negative regulatory circuits embedded in more complex networks. Altogether, these contributions laid the foundation for a wealth of studies demonstrating the versatility and power of logical modeling in molecular biology and beyond (see e.g., Thomas and D'Ari, 1990; Kauffman, 1993).

Briefly, in a logical model, each component is associated with a discrete variable, which is a logical (often Boolean i.e., binary) abstraction of its level of activity (or concentration). A logical function defines the next value of this variable, depending on the current levels of the regulators of that component. Such a model defines a discrete dynamical system where the state of the network (the component levels) evolves stepwise. Besides scalability (logical models with few hundreds components have been simulated), the appeal of this framework relies on its qualitative nature, as kinetic parameters and other precise knowledge about the molecular mechanisms at stake are not required. Despite this coarse grained abstraction, the resulting behaviors presumably capture the most salient properties of the modeled systems (Samaga and Klamt, 2013; Albert and Thakar, 2014; Le Novère, 2015). As a matter of fact, the logical framework proved useful in a wide range of biological applications: cell differentiation in developmental processes (for instance, drosophila development as in González et al., 2008; Sánchez et al., 2008; Fauré et al., 2014), haematopoiesis (Bonzanni et al., 2013), T lymphocyte activation and differentiation (see Section 5.1), cell cycle control (see Section 5.2) and more generally cell fate decisions such as proliferation, growth arrest, apoptosis, senescence, etc. (see e.g., Schlatter et al., 2009; Grieco et al., 2013; Mombach et al., 2014; Cohen et al., 2015).

Alternative modeling frameworks explicitly refer to sets of reaction rules (denoting molecular consumption and production processes) to model and analyze cellular networks (see Le Novère, 2015 for further details and references). In this respect, a logical model can be considered as an abstraction focusing on signed interactions denoting positive or negative influences between network components (defining the regulatory graph, which is completed by logical rules specifying the compositional effects of these influences). The logical framework is thus primarily used for signaling and gene regulation modeling.

For a general overview of the logical modeling of biological networks, we refer to existing reviews (Samaga and Klamt, 2013; Albert and Thakar, 2014; Le Novère, 2015; Naldi et al., 2015). Here, we emphasize the versatility of the logical formalism, as well as the relevance of a range of methods and tools. We first present formal definitions of (multi-valued) models and their associated dynamics, depending on a variety of updating schemes. As attractors and their reachability are of utmost interest when analyzing models of biological networks (see e.g., Huang et al., 2009), we particularly focus on approaches to determine model attractors and their reachability properties, as well as on the impact of variations of external signals on model behaviors. To demonstrate the relevance of the logical modeling and of the associated methodological advances, we survey several published logical models dealing with two important biological processes: (i) the activation and differentiation of T cells, and (ii) the control of cell proliferation.

The regulatory network controlling mammalian T helper (Th) lymphocyte activation and differentiation is of particular interest from the modeling point of view. First, this system has been largely studied experimentally, leading to the identification of many of the key molecular components involved. Furthermore, Th cell activation and differentiation are controlled by complex and intertwined intracellular signaling pathways and regulatory circuits, which ultimately enable the differentiation of Th cells into multiple functional subtypes, depending on the signals present in their microenvironment.

Also challenging and well studied are the networks controlling the initiation of cell division and the progression of cells along the main phases of mitotic cell cycles. Initially investigated in model organisms such as budding and fission yeasts, these networks have been deciphered in various other species, up to mammals. Models have been built to assess the implementation of the various cell cycle check points and the achievement of coordinated and robust oscillations in the activities of molecular components. Moreover, as defects of the cell cycle engine are one of the bases of cancer, many studies currently focus on mammalian cell cycle control networks.

In Section 2, we formally introduce the basics of the logical formalism and its main variants. The core of this paper demonstrates the assets of the framework with advanced methods and tools to analyze behavioral properties (Section 3), as well as to support data integration into models (Section 4). Finally, we illustrate these assets on T cell signaling and cell cycle control networks (Section 5).

## 2. Fundamentals of the logical formalism

We formally introduce the logical framework, defining models and their dynamics. Most common variants are presented, in particular regarding updating schemes and their impacts on dynamical properties. A selection of computational tools is then briefly presented.

### 2.1. Model definition

**Figure 1:**
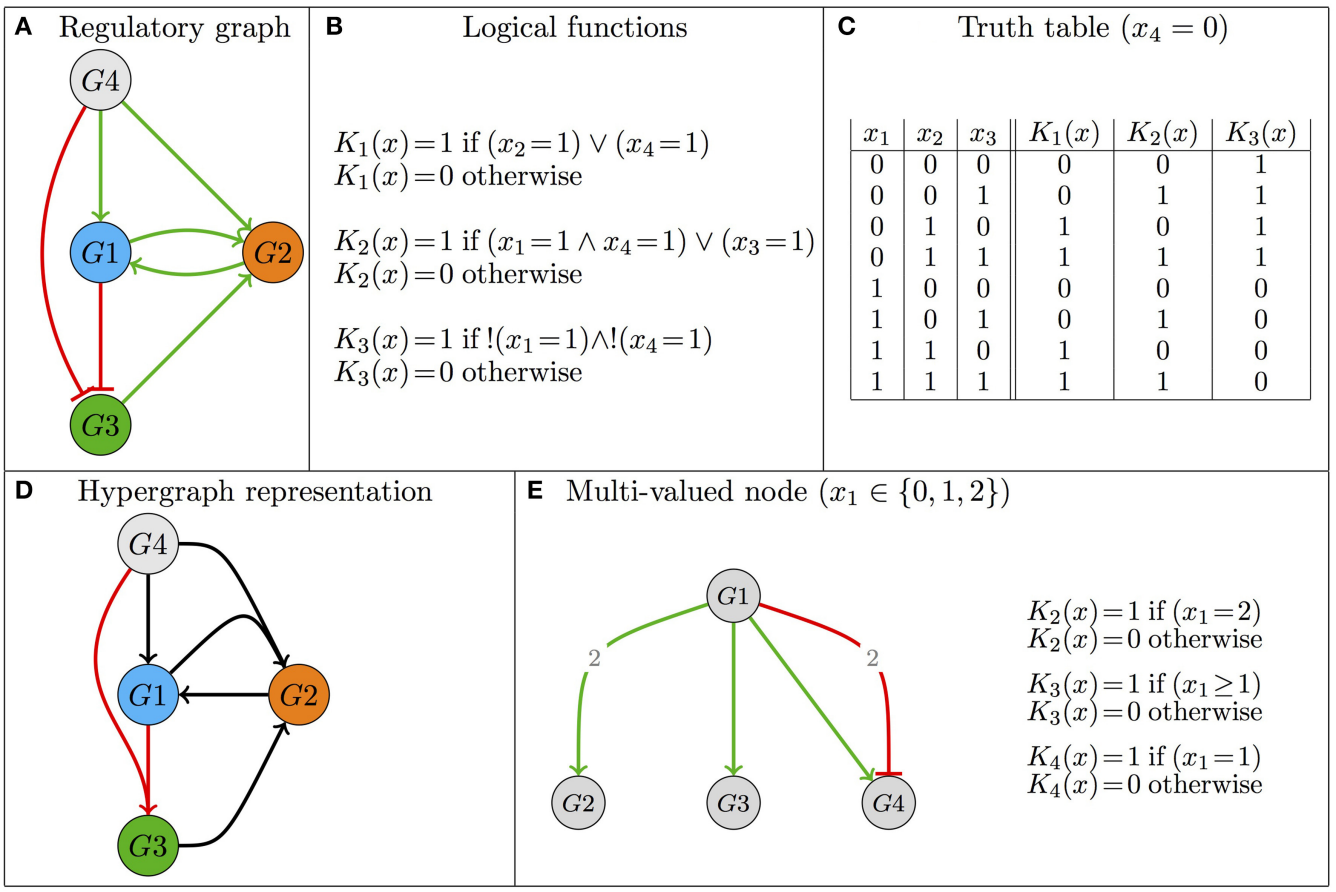
**Illustration of the basics of the logical formalism—Model definition**. **(A)** The regulatory graph defines the topology of the regulatory structure, where nodes denote regulatory components and edges represent regulatory effects (activations are denoted by green edges, whereas inhibitions are represented in red). **(B,C)** The evolution of the variables associated with the regulatory components is defined by the logical functions, which are written in the form of logical formulas or, alternatively, in the form of truth tables. “∧,” “∨,” and “!” stand for the logical operators AND, OR and NOT, respectively. Note that the regulatory graph in **(A)** can be recovered from the logical functions defined in **(B,C)**, but the reverse is not true (see main text). **(D)** Hypergraph as an alternative definition of the Boolean model of **(A–C)** (merged arrows denote AND operator). **(E)** Example motivating the introduction of a multi-valued variable; here *G*1 activates *G*2 and *G*3 at different thresholds and activates *G*4 when it is at level 1, but inhibits it at level 2 (see also Supplementary Figure S1).

The basic concepts presented in this section are illustrated in Figure 1. A logical model (G, K) of a regulatory network is defined by:

- A set of n regulatory components G = {*g*_1_, *g*_2_, …*g*_*n*_}, each *g*_*i*_ being associated with an integer variable, which takes its values in {0, …*max*_*i*_}, defining a discrete mapping of the range of the component functional levels (of activity or concentration). The (finite) state space S is defined as the cartesian product Π_*i* = 1, …*n*_{0, …*max*_*i*_} and a model state is thus a vector *g* = (*g*_1_, …*g*_*n*_).
- For each *g*_*i*_, a discrete function K_*i*_ defines its values, depending on the model states: K_*i*_ : S → {0, …*max*_*i*_}. The transition function K : S → S with K(*g*) = (K_1_(*g*), …K_*n*_(*g*)) thus defines the model behavior, but also the underlying regulatory graph (see below).

While a Boolean discretization is generally enough (i.e., *max*_*i*_ = 1 for all *i*), a regulatory component may operate at different levels on distinct targets, or yet, depending on its level, may have different effects on a given target. In such cases, it is necessary to consider a multi-valued variable whose maximal value is greater than 1 (see Figure 1E). Note that the discrete functions K_*i*_ are referred to as *logical functions*, even in the case of multi-valued variables. This denomination originates in Thomas and Snoussi's seminal work defining their *generalized kinetic logic* (Thomas and D'Ari, 1990).

The regulatory graph, denoted (G, R), is often available early on. It encompasses nodes denoting model components (regulatory components, elements of G), along with signed, directed edges, denoting regulatory activations or inhibitions (elements of R). The logical rules precisely encode these interactions. In other words, (G, R) can be deduced from K. Note, however, that several sets of logical rules can be compliant with a regulatory graph, which therefore defines a family of logical models.

There is a *functional* interaction from *g*_*j*_ to *g*_*i*_ (denoted (*g*_*j*_, *g*_*i*_) ∈ R) if and only if there exists a pair of neighboring states that only differ on the value of *g*_*j*_ and for which the function K_*i*_ takes a different value, thus indicating that a variation of *g*_*j*_ has an effect on the value of its target *g*_*i*_. More formally, assuming for simplicity that *g*_*j*_ is a Boolean variable, (*g*_*j*_, *g*_*i*_) ∈ R if and only if there exist two states *g* = (*g*_1_, …*g*_*j*−1_, 1, *g*_*j*+1_, …*g*_*n*_) and $\bar{g}=\left(g_{1},\ldots g_{j-1},0,g_{j+1},\ldots g_{n}\right)$ such that $K_{i}\left(g\right)\neq K_{i}\left(\bar{g}\right).$ Moreover, if $K_{i}\left(\bar{g}\right)<K_{i}\left(g\right)$, this interaction is an activation (because when *g*_*j*_ = 0 as in state $\bar{g}$, the function K_*i*_ defines a lower value for *g*_*i*_ than when *g*_*j*_ = 1 as in state *g*), otherwise it is an inhibition.

Specific classes of Boolean regulatory functions have been considered in the literature. The simplest specifies that a component is activated (its associated variable tends to 1) in the presence of at least one of its activators and in the absence of all of its inhibitors (e.g., Mendoza and Xenarios, 2006). Threshold networks constitute another popular class of Boolean models, in which the regulatory function is defined by comparing the (possibly weighted) sum of positive and negative regulatory contributions with a specific threshold (Li et al., 2004; Bornholdt, 2008). Finally, relying on the fact that any Boolean function can be written in a disjunctive normal form (a disjunction of conjunctive clauses, thus using exclusively the operators AND, OR and NOT), an alternative, refined representation uses hypergraphs (Klamt et al., 2009; Samaga and Klamt, 2013, Figure 1D).

### 2.2. Model dynamics

A logical model defines a discrete dynamics over its state space S. Given a state *g*, the transition function K specifies the possible changes of the model variables: if K(*g*) ≠ *g*, there is at least one variable *g*_*i*_ called to update toward the target value K_*i*_(*g*). Note that multi-valued variables are modified stepwise, i.e., if K_*i*_(*g*) differs from the current value of *g*_*i*_ by a value greater than 1, the next value of *g*_*i*_ is increased (or decreased) by 1. If K(*g*) = *g* then *g* is a stable state, in which each component value is maintained constant. Input components, which typically embody external signals, have no regulators and hence no associated logical rules. They are generally considered as being constant (their values representing a fixed environmental condition). However, how the model evolves upon input variations is of particular interest and is discussed in Section 3.2.

**Figure 2:**
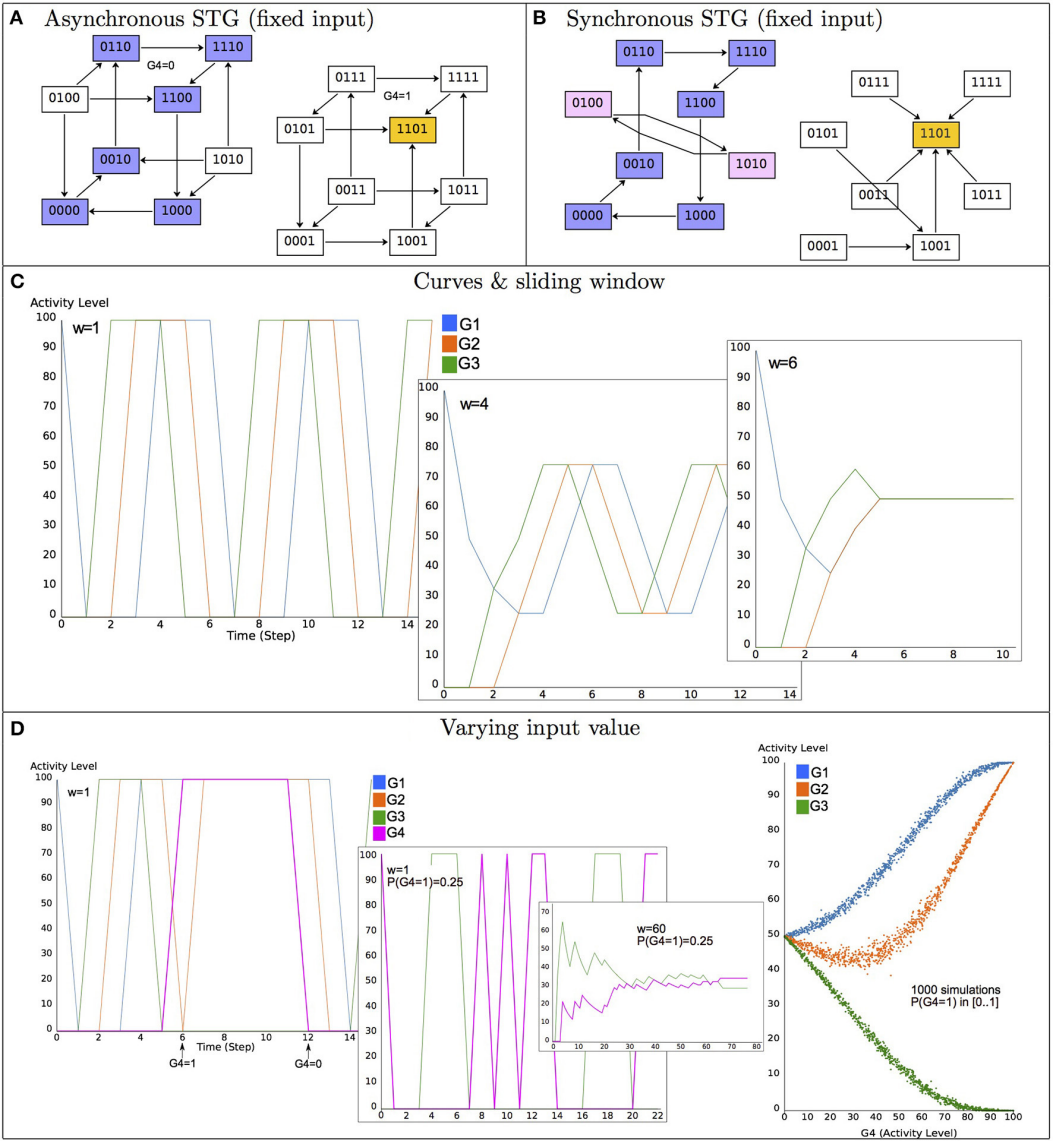
**Illustration of the basics of the logical formalism—Model dynamics**. **(A)** The asynchronous State Transition Graph (STG) of the model defined in Figure 1 **(A–C)**, with the input G4 maintained constant and concurrent transitions from states in which several variables are called to update their values. The yellow state 1101 (i.e., *x*_1_ = *x*_2_ = *x*_4_ = 1 and *x*_3_ = 0) is a stable state, the set of states in blue corresponds to a cyclic attractor. **(B)** The synchronous STG in which variables are simultaneously updated; the stable state is conserved, whereas a new terminal cycle appears (in pink). **(C)** Synchronous dynamics starting from the state 1000 and maintaining the input constant to 0 (activity levels are given in %, from 0 to 100%). For a sliding window of length *w* = 1 (see Equation 3), the curves conform the terminal cycle of **(B)** (in blue), the four variables oscillate between 0 and 1, with a period of 6; for *w* = 4, the mean values oscillate between 0.25 and 0.75; for *w* = 6, the mean values are constant to 0.5. **(D)** Illustration of the effect of different input variations (G4 value). When G4 is active with a probability 0.25, oscillations of the remaining components are altered (only G3 values are displayed, for legibility). The plot on the right shows the effect of varying the probability of G4 activity (from 0 to 1) on the mean values of the remaining components in the long term (i.e., in the attractor).

Model dynamics are conveniently represented in terms of *State Transition Graphs* (STG), where nodes denote states, while directed edges represent state transitions (Figure 2). Since the number of states is finite, model simulations always end up in a stable state or in a (potentially branched) cyclic trajectory. Stable states (devoid of transitions to other states) often represent cell differentiated states (cf. Section 5.1) or other kind of relevant, perduring situations. In contrast, cyclic trajectories may denote a biologically relevant periodic behavior, as in the case of cell cycle (cf. Section 5.2) or circadian rhythms. The mathematical counterparts of such asymptotic behaviors are called *attractors*, which are defined in the context of the logical formalism as terminal *Strongly Connected Components* (SCC) of the STG, i.e., maximal sets of mutually reachable states, with no transitions leaving the set. The set of states from which trajectories (exclusively) lead to an attractor is called its (strict) *basin of attraction*. Basins of attraction are particularly relevant since they define the reachable attractor(s) depending on the chosen initial state(s).

Dynamical properties of interest predominantly relate to the existence and reachability of the attractors. These are properties hard to assess in large models because the size of the state space (and thus of the STG) grows exponentially with the number of regulatory components. Section 3 presents several recent methods to identify attractors and to check their reachability properties.

If at state *g*, several variables are called to change their values (because their current values differ from the values returned by the corresponding logical functions), one has to specify how these changes should be performed. The two most common schemes are the *synchronous* and *asynchronous* updates. According to the first, all the variable updates are performed synchronously (i.e., simultaneously). Hence, the resulting deterministic dynamics defines, at each time step *t* (or iteration), the successor state of *g*(*t*):

(1) $$\begin{array}{l} g\left(t+1\right)=g_{i}\left(t\right)+signK_{i}\left(g\left(t\right)\right)-g_{i}\left(t\right){}_{i=1,\ldots n}, \end{array}$$

where *sign*(*p*) equals to 1 if *p* > 0, −1 if *p* < 0, and 0 otherwise. According to Equation (1), a successor state is defined by increasing or decreasing by 1 all the variables whose current values differ from the values specified by their logical functions. Note that if all the variables are Boolean, this equation can be written simply as *g*(*t* + 1) = K(*g*(*t*)). Given a state *g*, the synchronous update yields exactly one transition toward a successor state, which can be *g* itself, if all the variables are stable in *g*, i.e., K_*i*_(*g*) = *g*_*i*_, for all *g*_*i*_ ∈ G.

In contrast, with the asynchronous update, each variable is modified independently, yielding as many transitions (and successor states) as the number of updated variables (and hence potentially non-deterministic dynamics). At state *g*(*t*), for all *g*_*i*_ ∈ G such that K_*i*_(*g*(*t*)) ≠ *g*_*i*_(*t*), an asynchronous successor *g*(*t* + 1) of *g*(*t*) is defined as follows:

(2) $$\begin{array}{l} g_{i}(t+1)=g_{i}(t)+sign\left(K_{i}\left(g(t)\right)-g_{i}(t)\right),\\ g_{j}(t+1)=g_{j}(t)\text{ for all }j\neq i. \end{array}$$

Note that, according to this definition, a stable state has no successor. However, for any updating scheme, one may alternatively consider that a stable state is its own successor (with a self-loop transition).

In the context of asynchronous dynamics, priority classes, deterministic and stochastic schemes have been proposed, taking into account additional knowledge to penalize or discard unrealistic trajectories. Indeed, update classes can be defined, grounded on the nature of the processes involved, e.g., different time scales associated with transcriptional and post-translational processes (Chaves et al., 2005). At each time step (or iteration), the selection of updated variables is directed by their associated priority classes (Fauré et al., 2006), absolute ranks or probabilities (e.g., Albert and Thakar, 2014 and references therein). Generalizing the logical framework with a probabilistic interpretation, a finite Markov chain can be derived from the dynamics of a logical model. Considering the asynchronous update, Stoll et al. (2012) defined continuous or discrete time Markov processes by associating stochastic rates with the updates of the model components, and relied on a Gillespie algorithm to simulate the time evolution of component levels. This allows to get a more quantitative view of the model behavior (cf. Section 5.2). In Cell Collective, synchronous simulations also result in a Markov chain when the input components are associated with a probability (see Section 3.2 and Todd and Helikar, 2012).

When following a unique trajectory (defined by a synchronous update or selecting specific transitions among multiple asynchronous, concurrent trajectories), a natural alternative to the STG consists in displaying the evolution of the individual variables over time (see Figure 2C). To provide a different view of the model behavior, it has been also proposed to consider the mean values ${\tilde{g}}_{i}$ of a model variable *g*_*i*_ over a sliding window of (user-defined) length *w* (Helikar and Rogers, 2009):

(3) $$\begin{array}{l} \forall g_{i}\in G,\forall t\geq 0,{\tilde{g}}_{i}\left(t\right)=\frac{\sum_{0\leq k<min\left(w,t\right)}g_{i}\left(t-k\right)}{min\left(w,t\right)}. \end{array}$$

It is worth recalling that different updating schemes lead to different dynamics, thus impacting related properties (e.g., see Albert and Thakar, 2014). Briefly, compared to the synchronous scheme, asynchronous dynamics are more realistic in accounting for delays between updating orders and their executions. While stable states are the same for both the synchronous and asynchronous schemes, a striking example of how the resulting dynamics can differ is that of isolated regulatory circuits, for which the synchronous scheme leads to the appearance of additional cyclic attractors (Remy et al., 2003). Not only cyclical attractors may be different, but reachability properties are also modified. The asynchronous scheme generates concurrent trajectories, some of which are potentially unfeasible in regard to well-grounded choices between concurrent events. Hence, refined asynchronous schemes have been considered, such as priorities, fixed ranks or probabilities, which may also affect attractors and their reachability properties. Indeed, as some trajectories are preempted, transient oscillatory behaviors may be turned into cyclic attractors.

By way of conclusion, beyond the model definition as presented in Section 2.1, modelers need to specify an updating scheme and make this choice explicit when presenting their results. Moreover, model robustness could be assessed by probing different updating schemes and their impacts on attractors and their reachability properties.

### 2.3. A selection of computational tools

Here, we focus on the software tools used to generate results reported in the remaining sections. The web page http://colomoto.org/software/ provides a more comprehensive overview of available network modeling tools based on the logical framework.

**GINsim** (http://ginsim.org) supports the definition of multi-valued logical models, under the synchronous, asynchronous and priority updating schemes. Besides the explicit construction of STG (for reasonable sizes, i.e., in the order of a few million states), GINsim provides a number of methods to analyze model properties and supports model exports into various formats, in particular for model checking (see Section 3.1) (Chaouiya et al., 2012; Bérenguier et al., 2013).

**Cell Collective** (http://cellcollective.org) is a web-based software with a user friendly interface for model construction, simulation and analyses in a collaborative fashion. Its model repository provides a way for users to directly use and/or expand any of the 50 or so available models. Cell Collective supports Boolean models, considers synchronous updates, stochastic input simulations, and semi-continuous dose-response (input-output) analyses as shown in Section 5.2 (Helikar et al., 2012, 2013b).

**CellNetOptimizer** (CellNOpt, http://www.cellnopt.org) permits to define models of signaling networks as Boolean synchronous models. It further supports constrained fuzzy logic (Morris et al., 2011) and systems of differential equations (Wittmann et al., 2009). CellNOpt specificity is that, starting from a Prior Knowledge Network (i.e., a candidate topology of the signaling network under study), it creates a model by fitting its behavior to high-throughput biochemical data (MacNamara et al., 2012; Terfve et al., 2012).

**MaBoSS** (http://maboss.curie.fr) is a command-line tool simulating continuous/discrete time Markov processes induced by Boolean models (Stoll et al., 2012). Stochastic rates are associated with model component updates and a Gillespie algorithm is used to simulate the time evolution of component levels. Time evolutions of probabilities are estimated and global and semi-global characterizations of the whole system dynamics are further provided.

## 3. Model analysis

In this section we focus on a selection of methods to assess dynamical properties of logical models. Usage and relevance of these methods are illustrated in Section 5. We refer to Morris et al. (2010), Samaga and Klamt (2013), Albert and Thakar (2014), and Naldi et al. (2015) for further overviews.

### 3.1. Identifying the attractors and analyzing their reachability

As previously mentioned, properties of interest relate to attractors and their reachability properties. In small models (up to a dozen components), such properties can be easily recovered directly by constructing and analyzing the State Transition Graph (STG). However, for larger models, a variety of approaches based on different algorithmic techniques and efficient data structures have been proposed to handle the combinatorial explosion of the number of states.

Stable states, which do not depend on updating schemes, are relatively easy to identify because they correspond to the fixed points of the transition function. The algorithm implemented in GINsim relies on (multi-valued) decision diagrams to represent the (Boolean) stability function of each component *g*_*i*_ (which is true iff K_*i*_(*g*) = *g*_*i*_). Proper manipulations of this data structure enable the identification of all the stable states of a logical model of up to about hundred components (Naldi et al., 2007).

Identification of complex attractors is harder. Those are composed of several states and depend on the selected updating scheme (cf. Figure 2). In a synchronous dynamics, they correspond to terminal, elementary cycles (i.e., closed dynamical cycles in which each state has a unique successor), whose states are fixed points of the *p*^*th*^ iterate of K, for a cycle of length *p* (note that *p* is not known in advance). Hence, most existing methods sample or explorethe whole STG. Binary Decision Diagrams proved effective to perform such an exploration (Garg et al., 2008). Avoiding exploration of the state space, methods to identify stable subspaces (i.e., regions of the space space in which the model dynamics is trapped and thus contain attractors) have been recently proposed (Zañudo and Albert, 2013; Klarner et al., 2015).

Hierarchical Transition Graphs (HTG) have been defined as STG compactions revealing crucial properties of the dynamics (Bérenguier et al., 2013). Briefly, a HTG gathers (i) states that belong to the same SCC, and (ii) states that define trivial SCCs (i.e., if reached once, they cannot be revisited) and from which the same set of attractors and SCCs can be reached (cf. Supplementary Figure S1). Hence a HTG provides an informative view of the dynamics in terms of attractors and their basins of attraction.

To quantify attractor reachability, Mendes et al. (2014) presented Avatar, a Monte Carlo simulation algorithm adapted to speed up exit from transient cycles and to identify complex attractors if those are not known beforehand. Avatar allows to estimate the probability of reaching an attractor from an initial state or from any initial state (i.e., sampling the state space) under the assumption of equiprobability of concurrent transitions. In turn, MaBoSS, mentioned in Section 2.3, provides an estimation of state probabilities over time (cf. Section 5.2), along with further characterizations of the whole dynamics.

Model checking was proposed in the early 1980s to verify a (set of) specification(s) against very large models of hardware and software systems. Since then, methodologies have been improved as well as their ranges of applicability. Notably, in the mid 2000s, model checking started to be applied in Systems Biology, mainly to verify qualitative systems dynamics (e.g., Chabrier and Fages, 2003; Batt et al., 2005; Arellano et al., 2011; Abou-Jaoudé et al., 2015), but also for hybrid systems considering continuous time or continuous state variables (e.g., Hinton et al., 2006; Clarke et al., 2008; see also Brim et al., 2013 for an overview). A model checker verifies whether a model of a system satisfies a set of properties, answering true/false for each property. The dynamics is represented as a specific transition system and properties are specified by temporal logic formulas. Different temporal logics exist, each with specific operators to explicitly reason about time or about precedence relationships between states. The temporal logics mostly used relate to the latter: the Linear Time Logic (LTL), in which time is considered linear, and the Computation Tree Logic (CTL) in which alternative time lines are considered (Clarke et al., 1999).

In the asynchronous dynamics of a logical model, a state may have multiple successors and hence lead to alternative paths, which makes CTL particularly useful. To check reachability properties, as illustrated in Section 5.2, we use CTL temporal operators, with the following syntax and semantics (see Clarke et al., 1999 for a complete reference of CTL operators):

- EF(Φ), there is at least one path leading to a state satisfying the property Φ;
- E[ΨUΦ], there is at least one path satisfying Ψ until it reaches a state satisfying Φ.

In the verification of software/hardware systems, a property is true if and only if it is true for every state in the set of initial states. However, when verifying biological systems, one is often interested in the existence of a reachability path from at least one of the initial states. The solution lies in the specification of the negated property (i.e., absence of reachability), which forces the model checker to answer false if there is at least one reachability path (used in Section 5.2).

A popular model checker is NuSMV (Cimatti et al., 2002). GINsim provides an export into a NuSMV description with the model rules, updating scheme and a (set of) initial state(s), together with other optional parameters. In a NuSMV description, the (set of) initial state(s) is specified using the keyword INIT, and the (set of) properties is specified using the keyword SPEC (cf. Section 5).

### 3.2. Assessing model behaviors upon input variations

Recall that input components have no associated regulatory function and are thus generally kept constant throughout simulation. This means that there are no transitions between states of the STG differing on values of input components (see Figure 2). However, these disconnected STG sub-graphs can be connected by adding bi-directional transitions, which account for unconstrained variations of the input components. Using model checking tools, it is then possible to check properties for which inputs freely vary along a simulation. In order to account for a distinct semantics of inputs and internal (regulated) components, the Action Restricted Computation Tree Logic (ARCTL) is used (Lomuscio et al., 2007; Monteiro and Chaouiya, 2012). ARCTL extends CTL, imposing an additional path restriction on a subset of inputs while letting the remaining inputs to freely vary. This temporal logic was implemented in NuSMV-ARCTL, which extends NUSMV. In Section 5.1, we take advantage of a subset of ARCTL operators with the following syntax and semantics (see Lomuscio et al., 2007 for a complete description of ARCTL operators):

- EAF(α)(Φ), there is at least one path leading to a state satisfying Φ, and the input restriction α must be satisfied along this path;
- AAG(α)(Φ), all the states of all paths must satisfy Φ, and the input restriction α must be satisfied along these paths.

Other approaches have been developed to simulate Boolean models under stochastic and continuous environments (Helikar and Rogers, 2009; Helikar et al., 2012). Considering a synchronous update, a model input can be allocated a probability to be in its active state at each simulation step (see Figure 2D). This probability may represent finer levels of external signals. Furthermore, once a Boolean network has reached an attractor, the average active/inactive states of each component over the entire attractor can be calculated providing a characterization of the component activity level in this attractor (Todd and Helikar, 2012). Varying continuously the probabilities of input states (from 0 to 1), input-output dose-response (titration) curves can be generated, similar to those traditionally produced in experimental studies, for example to study the effects of different concentrations of a drug (Madrahimov et al., 2013) or of different concentrations of receptor ligands as in **Figure 6** (Helikar et al., 2013a). Currently, this approach is supported by the Cell Collective (cf. Section 2.3), and by the stand-alone command-line simulation engine, ChemChains (Helikar and Rogers, 2009).

### 3.3. Model reduction

A natural solution to lessen the combinatorial explosion issue is to reduce the size of the model. Any reduction potentially alters the properties of a model by modifying its dynamics. However, when the reduction impacts on the dynamics are well mastered, the analysis of a reduced model can be used to deduce interesting properties of the original model. This is the case of the reduction method that removes components while properly modifying the logical functions of their targets, which thus become directly affected by the regulators of the removed components (Naldi et al., 2011, 2012; Saadatpour et al., 2013). As a consequence, a self-regulated component cannot be removed, firstly because this definition is not applicable, but also because regulatory circuits are known to drive important dynamical properties and thus should not be concealed (Thieffry, 2007). The key point about this reduction is that it does not generate novel trajectories and thus reachability properties that are verified in the reduced model are also true in the original model. Furthermore, Naldi et al. (2011) demonstrated that all the stable states and elementary cyclic attractors of the asynchronous dynamics are preserved. Because transitions of the original STG may be discarded (removing a component amounts to consider that its evolution is faster than that of the concurrent components), more complex attractors may be split in two or more complex attractors, while transient SCC may become terminal. However, Saadatpour et al. (2013) showed that, for constant input values, all the attractors are preserved when reducing input and pseudo-input components (i.e., components that are only regulated by inputs or by pseudo-inputs), as well as mediator components (which are characterized by a unique regulator and a unique target). Furthermore, both attractors and reachability properties are preserved when reducing output and pseudo-output components (i.e., components with no target, or whose targets are only outputs or pseudo-outputs, see Naldi et al., 2012).

### 3.4. Perturbation analyses

In the logical framework, it is straightforward to define perturbations. Perturbations affecting model components often merely amount to force the corresponding variables to take specific values. For example, to specify a knock-out, it suffices to set the variable to 0, whereas for an ectopic expression the variable is set to its maximal value. Stimulation of a signaling pathway at the receptor level can be simulated by setting the variable describing the receptor to 1, and blockage with a drug of a protein by setting it to 0. By modifying the regulatory functions, subtler perturbations can be defined as, for example mutations in a promoter region, turning a component insensitive to a given regulator (cf. Section 5.2).

## 4. Model and data integration

### 4.1. Integration of experimental data

Because logical models provide a flexible framework to encode different biological events, with various granularities, they are particularly well suited to examine experimental data. Perturbations (genetic alterations, treatment with drugs or ligands, etc.), can be easily encoded in the model (cf. Section 3.4), and simulation results can then be mapped to the measured values of specific biological components upon these perturbations.

Different types of experimental data have been integrated within logical models. Genetic data are commonly used to define models and simulations (e.g., mutations or knockdown conditions), for various model organisms, from microbes (Thieffry and Thomas, 1995), to cancer (Remy et al., 2015), and many others. The data type used as readout depends on the system under study. In the case of gene regulatory networks, gene expression data are typically used, while for signal transduction, protein phosphorylation data are normally used. It is also possible to include non-molecular data, such as phenotypic measurements like growth, which is useful e.g., to connect the effect of a drug on a signaling pathway with its effect on cellular growth (Kirouac et al., 2013; Flobak et al., 2015).

The integration of experiments and model can occur at different levels: (i) *a priori* in the building phase, to define or refine the model, (ii) *a posteriori*, to fit a generic model and obtain a model specific to certain conditions, and (iii) to (in)validate a model by challenging it to predict experimental data under specific conditions.

Model fitting to data allows to refine a given model structure relying on dedicated experiments. Because general network information is often not cell or context specific, such refinements lead to models that describe more accurately specific cellular situations. Such model adjustment can be done manually, by iteratively changing the model and testing how well the resulting model matches experimental data. For high-throughput data sets, this process has been automatized by casting it as an optimization problem (Saez-Rodriguez et al., 2009). This methodology can be applied in multiple biological contexts and to different types of data. In the case of signaling, as stated above, proteomic data are particularly adequate and can be obtained with antibody based platforms, such as protein arrays or luminex (Saez-Rodriguez et al., 2009), or using mass spectrometry (Terfve et al., 2015). Gene expression data can also be used (Crespo et al., 2013; Keller et al., 2016).

In addition to using experimental data for logical model construction, various types of data available in many databases can be exploited, in turn, to interpret simulation experiments and further validate the models and associated predictions. The advantage of dynamical models is that they can generate hypotheses about any targeted component, or about the system as a whole. For example, Puniya et al. (2016) interrogated a comprehensive signal transduction network model under all possible knock-in and knock-out perturbations, resulting in the identification and ranking of the most and least influential model components. These components were further mapped on various databases, resulting in the prediction of a new combinatorial drug target in a cancer setting.

In practical terms, standardized names and proper annotations using controlled vocabularies are essential for a correct integration of models and data. This issue is discussed in the next section.

### 4.2. Exchange formats and model documentation

As the popularity of logical modeling increases, standardization issues have to be tackled. To this intent, the informal consortium CoLoMoTo (http://www.colomoto.org) gathers researchers developing logical models, methods and tools (Naldi et al., 2015). The definition of a common file format was identified as a primary requirement to allow model exchange and software interoperability. Model encoding in a standard format facilitates model reuse for extension or composition. In the context of SBML Level 3 (Systems Biology Markup Language Hucka et al., 2003), the SBML Qual (for qualitative) package has been defined to store logical models (Chaouiya et al., 2013, 2015). This format is currently supported by a number of software tools, including GINsim, Cell Collective, CellNOpt, the tools mentioned in this paper. Hence, models stored in the SBML qual format can be exchanged between these tools. Thanks to the LogicalModel library, GINsim also provides an export of Boolean models to MaBOSS, in addition to several other formats (Chaouiya et al., 2013).

To allow reproducibility of *in silico* experiments, simulation settings must be specified along with the model itself. These settings include the initial condition(s) and a precise description of the updating scheme. Model perturbations may be also considered as specific simulation settings. For example, the software GINsim allows to store all this information in the form of a set of parameter settings (or simulation scenarios). Cell Collective stores simulation settings in a database. The Simulation Experiment Description Markup Language (SED-ML) has been defined as a standard format for encoding simulation experiments (Waltemath et al., 2011). One objective of CoLoMoTo is to promote the use of such a format, possibly by extending it to support specificities of logical modeling.

Proper documentation and annotation are crucial for reuse and expansion of computational models by the community. Often, published models lack information (evidence and/or clear assumptions) documenting model components, interactions and rules. Several efforts already exist to address this issue. The Minimum Information Requested In the Annotation of biochemical Models (MIRIAM) (Le Novère et al., 2005) was developed to standardize the type of information (e.g., connections to controlled vocabularies as well as to various databases) that should be included as model metadata. While MIRIAM (and other standards) provides minimal guidelines to ensure model reproducibility, additional efforts are needed to increase the overall quality (breadth and detail) of model documentation. For instance, modelers and curators can provide detailed and exhaustive evidences supporting model components and interactions when available, or assumptions in the case of unavailable experimental observations. This is facilitated in Cell Collective, which provides a Knowledge Base for each model.

Finally, BioModels database (Chelliah et al., 2015) and other model repositories such as those of GINsim and Cell Collective are also essential to ensure that models are available to the community for reproducibility of the results as well as for model reuse.

## 5. Logical modeling and analyses of two distinctive applications

### 5.1. Application 1: T cell signaling

T lymphocytes play a central role in the adaptive immune response in mammals. Cytotoxic CD8+ T cells kill cells infected by viruses or malignant cells, whereas CD4+ T helper (Th) cells orchestrate the function of a large diversity of effector immune cells (including B cells, macrophages, granulocytes, and NK cells) (Murphy et al., 2012). Activation of T cells and their subsequent differentiation into effector or regulatory cells result from the integration of a large panel of signals from their microenvironment. Initially in a naïve state, T cells are activated by three main types of signals: (i) T cell receptor (TCR) activation, through the specific recognition of foreign antigens presented by antigen presenting cells (APCs), (ii) co-inhibitory and co-stimulatory signals, and (iii) cytokines. The integration of these multiple signals initiates a plethora of signaling cascades, regulating complex and intertwined networks, which ultimately control T cell activation, proliferation and differentiation into effector or regulatory cells expressing specific markers.

For example, Th1 subtype is characterized by the production of interferon gamma (IFN-γ), leading to the clearance of intracellular pathogens, whereas Th2 cells secrete the cytokines interleukin-4 (IL-4), IL-5 and IL-13, involved in the elimination of helminths. Recently, additional Th subsets (e.g., Th17, Treg, Tfh, Th9, Th22) have been characterized. Furthermore, recent experimental evidences emphasize the diversity and plasticity of T cells, challenging the classical picture of irreversible branching differentiation (Nakayamada et al., 2012).

In order to decipher the mechanisms underlying T lymphocyte activation and differentiation, various logical models have been proposed, each addressing specific aspects (cf. Table 1). Hereafter, we discuss a sample of these modeling efforts to emphasize specific aspects of modeling and analysis, as well as insights into the regulation of T cell activation and differentiation.

**Table 1:** **Selected logical models of T cell signal transduction and gene regulation**.

**Publication**	**Characteristics (nb components)**	**Dynamics**	**Availability**
Mendoza, 2006	Multilevel model; CD4+ T cell differentiation regulatory network (17)	Stable state analysis, perturbation analysis, circuit analysis	GINsim
Saez-Rodriguez et al., 2007	Boolean model; T cell receptor signal transduction network (94)	Stable state analysis, input and perturbation analysis	Cell Collective
Zhang et al., 2008; Saadatpour et al., 2011	Boolean model; T cell survival signal transduction network (60)	Asynchronous update, perturbation analysis, model reduction, attractor identification	GINsim Cell Collective
Naldi et al., 2010	Multilevel model; CD4+ T cell differentiation regulatory network (65)	Asynchronous update, model reduction, circuit analysis, Th cell plasticity	GINsim
Martínez-Sosa and Mendoza, 2013	Boolean model; CD4+ and CD8+ T cells regulatory network (50)	Synchronous update, attractor analysis, perturbation analysis	Cell Collective
Miskov-Zivanov et al., 2013	Multilevel model; TCR signaling pathways (38)	Random asynchronous update, introduction of delays, duration of input stimuli modeled as a number of updating rounds	Cell Collective
Conroy et al., 2014	Boolean model; TCR and integrin signaling network and T cell differentiation regulatory network (188)	Synchronous update, stochastic inputs, perturbation effects on downstream components	Cell Collective
Oyeyemi et al., 2015	Boolean model; HIV-T cell interaction network (137)	Stable state analysis, perturbation analysis	Cell Collective
Abou-Jaoudé et al., 2015	Multilevel model; CD4+ T cell differentiation regulatory network (101)	Asynchronous update, model reduction, stable state analysis, model checking, Th cell plasticity	GINsim
Martinez-Sanchez et al., 2015	Boolean model; CD4+ T cell differentiation regulatory network (85)	Model reduction, attractor analysis, perturbation analysis, Th cell plasticity	Cell Collective BioModels DB (non-curated branch)

Relying on the initial identification of Th1 and Th2 dichotomy, Mendoza (2006) proposed a logical model of the differentiation network accounting for some aspects of Th commitment toward these two cell types. The author could capture Th1 and Th2 cellular types in terms of stables states of the model, and got further insights into the intracellular circuits involved in the delineation of the corresponding basins of attractions. Naldi et al. (2010) extended Mendoza's model to cover additional signaling pathways and Th subsets (Th17, Treg), using GINsim for model construction and analysis. As the model was too large for a direct analysis of its dynamics, the authors applied a reduction method (cf. Section 3.3), which led to a model encompassing 34 components, amenable to analysis through systematic simulations. Following the identification of all the stable states, these were grouped according to relevant phenotypic Th markers, abstracting away input values. The model accounts for the canonical Th1, Th2, Th17, and Treg subtypes, as well as for additional hybrid Th subtypes coexpressing combinations of canonical Th markers. Finally, the authors assessed the stability of the identified Th subtypes, under specific polarizing environmental conditions (defined by model input values), by iterating rounds of simulation of the reduced model dynamics. Interestingly, this reachability analysis emphasized the plasticity of the Th subtypes upon environmental changes, with some cell types predicted to be highly labile (Th17, Treg) whereas other are shown to be more robust (Th1, Th2).

**Figure 3:**
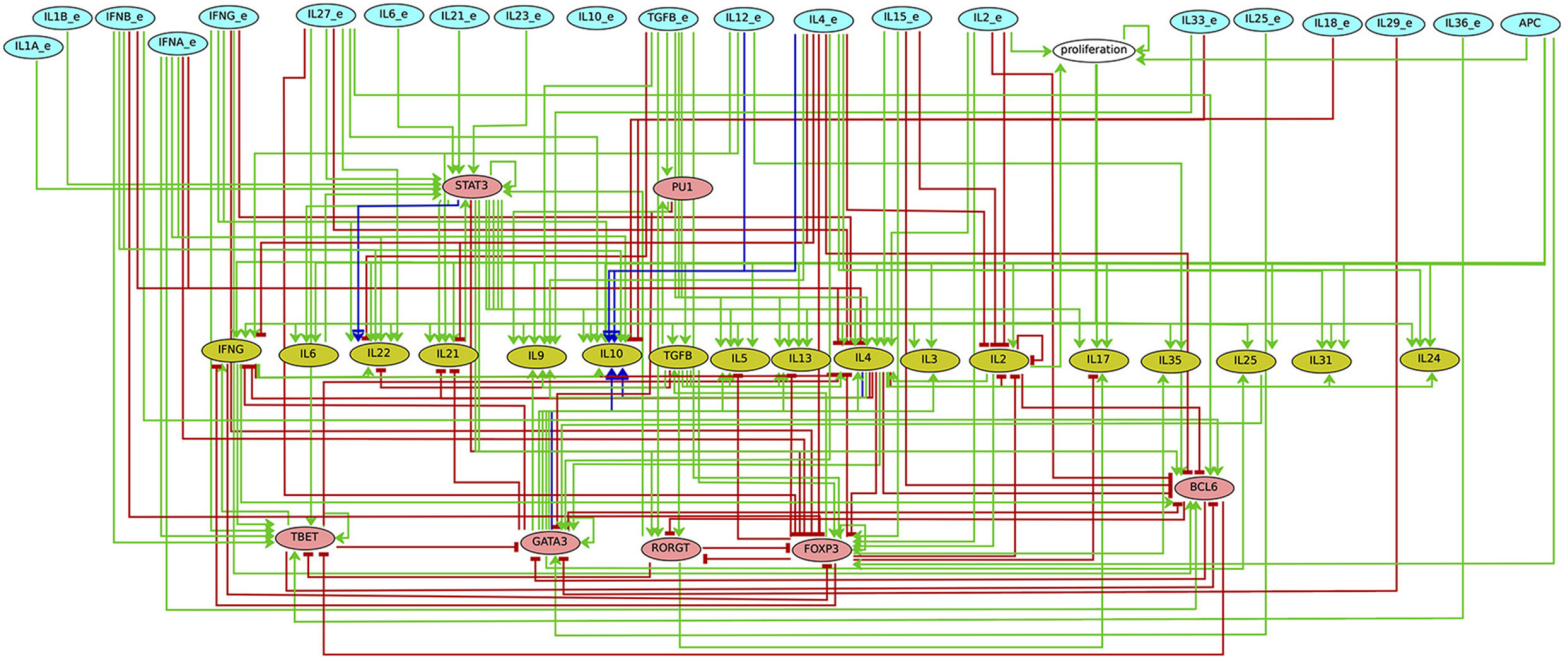
**Regulatory graph of the reduced version of the Th differentiation logical model in Abou-Jaoudé et al. (2015)**. The reduced model encompasses 46 nodes (among which 21 inputs) instead of 101 nodes in the original one. The components denoting the inputs are in blue, those representing the secreted cytokines in olive. Pink nodes denote transcription factors. Green edges denote activations whereas red blunt ones correspond to inhibitions. Blue edges represent dual interactions.

Extending this model, Abou-Jaoudé et al. (2015) proposed a multi-valued model accounting for novel canonical Th subtypes, namely Th9, Th22, Tfh, with the integration of additional transcription factors (e.g., PU.1, Bcl6) and cytokine pathways involved in Th cell commitment. Following the approach of Naldi et al. (2010) and considering a reduced version of the model (cf. Figure 3), all the stable states were identified and grouped according to phenotypic markers, thereby defining expression patterns associated with each canonical Th subtype. This analysis allowed to capture the novel canonical subtypes and predicted hybrid subtypes in terms of stable states. Noteworthy, the interpretation of the input dependency of the stability of these states is hindered by the gigantic number of input configurations (2^21^ value combinations of the 21 binary inputs). To cope with this combinatorial explosion, one can further cluster these stable states according to relevant input signatures.

Abou-Jaoudé et al. (2015) used model checking to efficiently analyze Th cell plasticity under relevant polarizing conditions. More precisely, using NuSMV-ARCTL (cf. Section 3.2), reachability properties between the canonical Th subtypes were systematically analyzed, considering relevant cytokinic environmental conditions. The following generic ARCTL property was specified to verify the existence of a reachability path from a canonical Th pattern *c*_1_ toward a (stable) canonical Th pattern *c*_2_ under an input condition *e* (the & operator denotes the conjunction):

$$\begin{array}{l} \text{INIT}c_{1};\text{SPEC EAF}\left(e\right)\left(c2\&\text{AAG}\left(e\right)\left(c_{2}\right)\right). \end{array}$$

**Figure 4:**
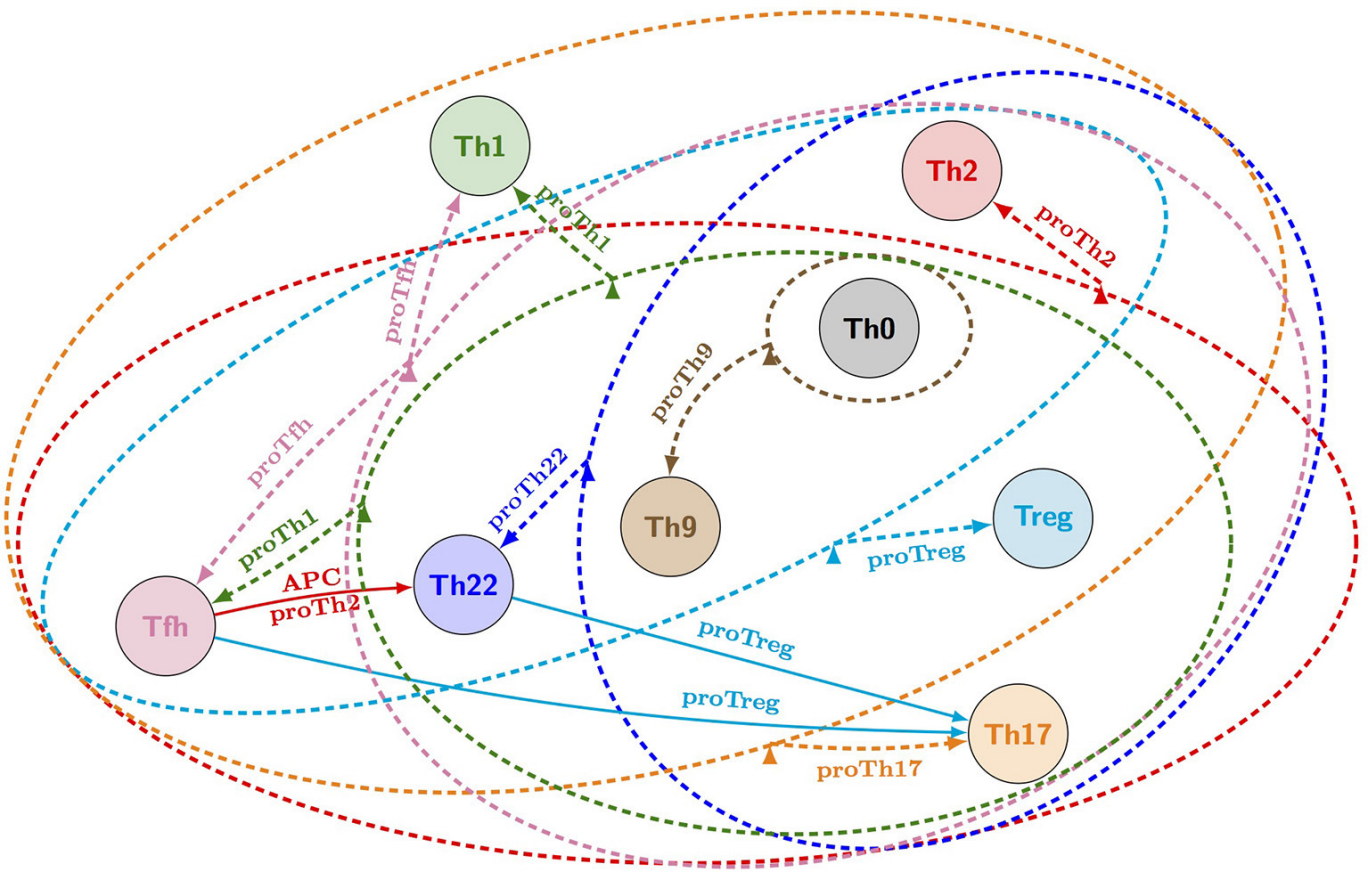
**Reprogramming graph considering the canonical Th subtypes (generated with the model checker NuSMV-ARCTL; adapted from Abou-Jaoudé et al., 2015)**. Ellipses gather all subtypes that, under the same environmental condition, differentiate toward a particular stable subtype (defined in Table 3 in Abou-Jaoudé et al., 2015). Dashed arrows connect ellipses to a (set of) differentiated state(s) and are labeled with the corresponding environmental conditions. Solid arrows denote specific reachability conditions between pairs of subtypes, under a particular environmental condition. Colors of arrows and ellipses indicate the environmental conditions of the corresponding subtype color. For example: from Th2, Th22, Th9, Th0, Treg, and Th17 subtypes (gathered in the pink ellipse), a “proTfh” condition leads to reprogramming into both Tfh (pink node) and Th1 subtypes; while from Th22, a “proTreg” condition leads to reprogramming into both Th17 and Treg subtypes.

Results were synthetically represented in the form of a reprogramming graph, which reproduces various polarizing events experimentally observed and uncovers many reprogramming scenarios between Th subtypes (see Figure 4). In particular, several strategies allowing Th1 vs. Th2 interconversions could be identified, in accordance with recent experimental observations challenging Th1-Th2 dichotomy (Antebi et al., 2013).

**Figure 5:**
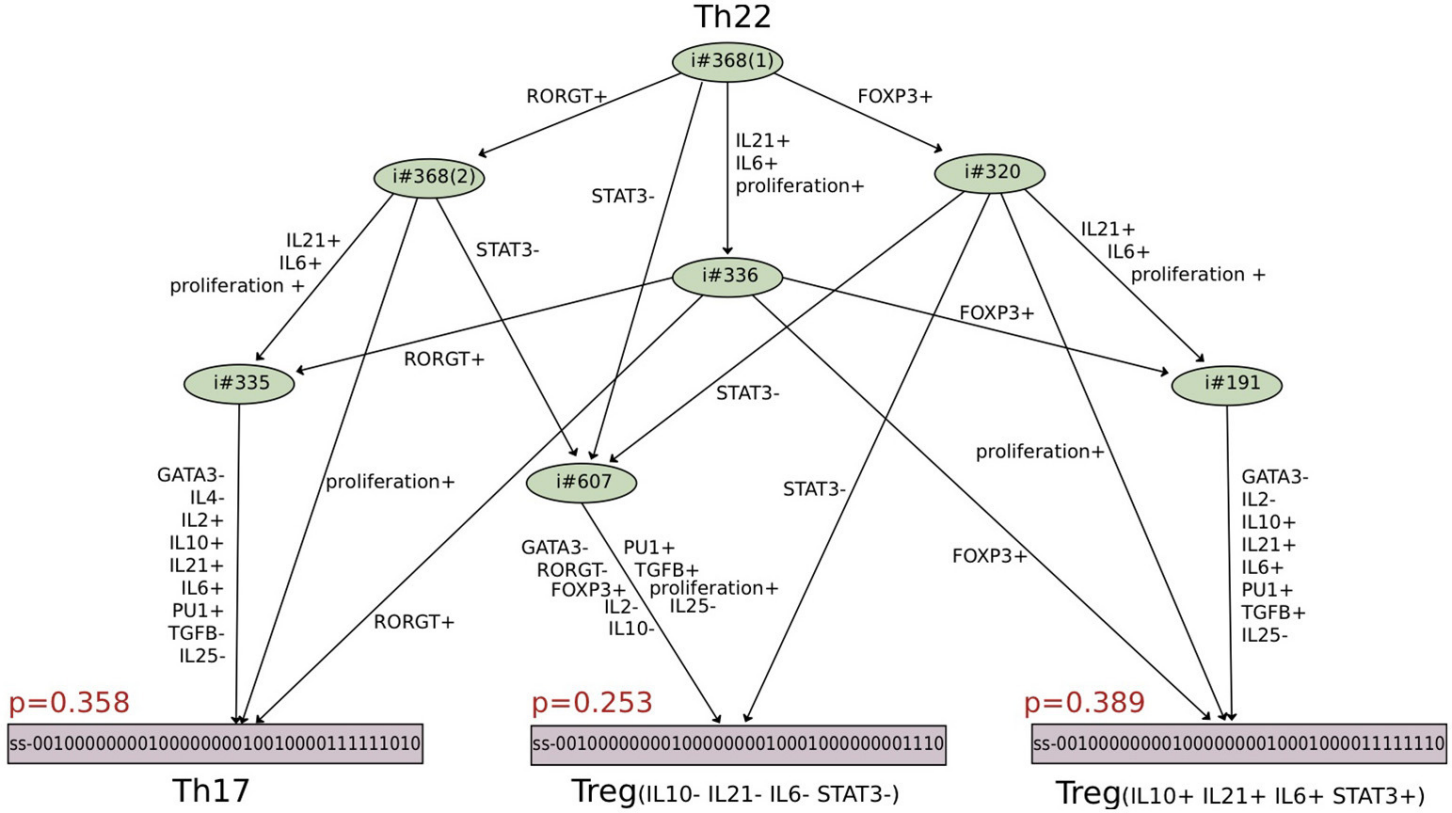
**Hierarchical Transition Graph (HTG) generated with GINsim considering an asynchronous simulation of the model shown in Figure 3 (Abou-Jaoudé et al., 2015)**. The bottom nodes correspond to the stable states, which are reachable starting from the initial conditions corresponding to the set of states characterizing Th22 cell type, under a Treg polarizing environment (upper node). The states reachable from the initial conditions, except the stable states, are grouped together into irreversible transient components (in green), the symbol ♯ precedes the number of states composing these nodes. The HTG encompasses 10 nodes (in contrast with the 2528 states of the corresponding STG). The labels associated with the arcs highlight the crucial transitions involved in the choice between the attractors (see Supplementary Figure S1). Each stable state is annotated with the probability in red of being reached from Th22 subtype under the Treg polarizing condition, considering 1000 simulations (computed with the software Avatar). The components are ordered as follows: first the external input cytokines IL1B, IFNG, IL2, IL4, IL6, IL10, IL12, IL15, IL21, IL23, IL27, TGFB, IL36, IL33, IL18, IL25, IFNB, IFNA, IL1A, IL29, followed by the component representing the Antigen Presenting Cells, then the transcription factors TBET, GATA3, RORGT, FOXP3, BCL6, followed by the secreted cytokines IFNG, IL4, IL2, IL10, IL21, IL6, followed by the transcription factors STAT3 and PU1, then the secreted cytokine TGFB, followed by a node denoting the proliferation of Th cells and finally the secreted cytokine IL25.

Other scenarios where a Th subtype can follow distinct fates under the same environmental conditions were also unraveled by this analysis. To get comprehensive insights into the alternative trajectories underlying different cell decisions, a HTG representation of the dynamics can be used. Figure 5 provides an example of such a representation starting from Th22 cells and immersing them into a Treg polarizing environmental condition. We see here that three stable states can be reached, one corresponding to a Th17 cell type, and two corresponding to Treg cell types. The cell decision between these phenotypes mainly depends on the concurrent activation of Rorgt (the master regulator of Th17 cells) and Foxp3 (the master regulator of Treg cells). Further insight into the reachability of the three attractors can be extracted by performing a reachability analysis with the Avatar algorithm, quantifying the reachability probability of each attractor (Mendes et al., 2014). A thousand Avatar simulations were enough to observe a stabilization of the reachability probabilities of the three stable states. These indicated a higher probability to reach the Treg stable states (0.642) than the Th17 state (0.358), suggesting that a Treg environment would favor Th22 cells reprogramming toward a Treg rather than a Th17 phenotype (Figure 5).

Other modeling works have focused on the signaling pathways underlying T cell activation, survival and proliferation. Saez-Rodriguez et al. (2007) established a Boolean model of T cell activation following the engagement of TCR and co-stimulatory receptor CD4 and CD28, using CellNetAnalyzer for model definition and analysis. Here, an analysis based on steady-state approximation was used. The reasoning being that in signal transduction several different time scales operate; a first wave of activation occurs upon stimulation with ligands and drugs, which often takes only a few minutes, and this is followed by feedback processes, which are typically slower. This approximation is clearly not accurate, but it permits the consideration of large networks in a simple and efficient manner. The model was able to recapitulate a large number of published data in both wild-type and knock-out conditions, as well as to predict unexpected signaling patterns after specific stimulation of the co-receptor CD28 and knock-out of the kinase Fyn, which were subsequently experimentally validated (Saez-Rodriguez et al., 2007).

**Figure 6:**
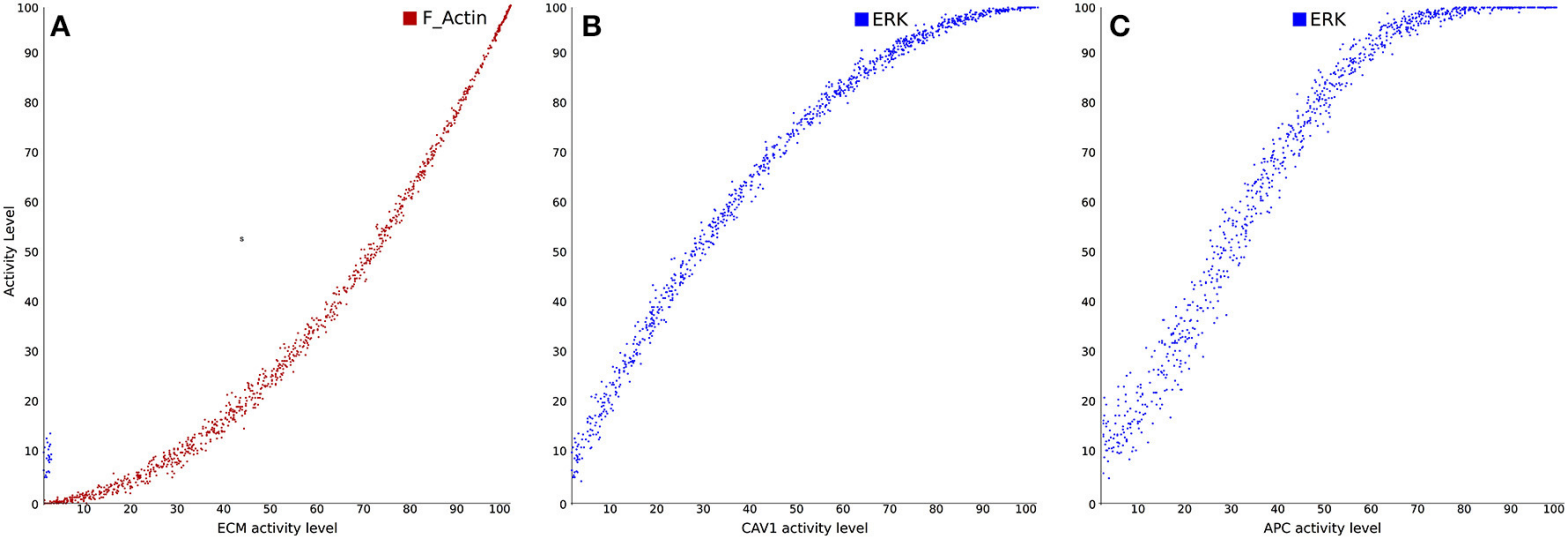
**Examples of dose-response analyses in a signal transduction and gene regulatory model in Cell Collective (adapted from Conroy et al., 2014)**. **(A)** Stimulation of filamentous actin polymerization in response to varying levels of cellular interaction with extracellular matrix (ECM). **(B)** Stimulation of the mitogen-activated protein kinase (MAPK) pathway in response to Cav1 activation. **(C)** Activation of the MAPK pathway in response to stimulation by antigen-presenting cells (APC).

Finally, several logical models were proposed to analyze T cell signaling networks in pathogenic situations, in particular in the context of T cell leukemia, a disease characterized by an abnormal proliferation of T cells (Zhang et al., 2008; Saadatpour et al., 2011; Conroy et al., 2014). Specifically, Conroy et al. (2014) developed a logical model to better understand the role of caveolin-1 (Cav1; an important regulator of endocytosis) in T-cell leukemia. Figure 6 illustrates input-output simulations and analyses demonstrating the ability of the model to correctly reproduce previously described and documented relationships between different components of the modeled network. Besides, the model allowed to identify the protein products most affected by CAV1+∕+, CAV1+∕−, and CAV1−∕− under immunocompetent and immunocompromised conditions. Simulation results suggested that CAV1 expression regulates Ras-related C3 botulinum toxin substrate 1 (RAC1), B-cell lymphoma/leukemia 10 (BCL10), GATA-binding protein 3 (GATA3), CD26, and CD28. In addition to validating these predictions in Cav1 knock-out mice, model results were further successfully validated against gene expression signatures obtained from the Gene Expression Omnibus (GEO) database.

### 5.2. Application 2: cell cycle control

Tightly controlled by a sophisticated regulatory network involving transcriptional regulations and protein modifications, cell proliferation involves successive phases governing genome replication (S phase) and cell division (mitosis or M phase), separated by regulated irreversible transitions (checkpoints). The main components and regulatory interactions controlling cell cycle were initially identified in simplified model systems, including fission and budding yeasts, as well as early Xenopus zygotic mitoses. The underlying core networks have been modeled using differential equations, leading to novel insights into their organization and dynamical properties (see Ferrell et al., 2011; Tyson and Novák, 2015 for recent reviews). However, extension and analysis of such differential models become really difficult as the number of experimentally identified components and interactions increases. This led several groups to consider Boolean or more sophisticated logical formalisms to build comprehensive models of cell cycle control networks (Table 2). Cell cycle networks present particular difficulties from the point of view of logical modeling. On the one hand, cell cycling behavior tentatively corresponds to a cyclic attractor, or at least to some multiple state pathway in the STG (rather than to a logical stable state as for the Th subtypes mentioned above), which are hard to compute. On the other hand, of most importance is the precise succession of component switches along the cell cycle, ensuring the proper temporal articulation of the molecular processes required for successful genome replication and repartition, along with timely and balanced cell division.

**Table 2:** **Selected logical models of cell cycle networks in different organisms**.

**Publication**	**Organism**	**Characteristics (nb components)**	**Dynamics**	**Availability**
Li et al., 2004	Budding Yeast	Boolean model; Threshold logical functions (11)	Synchronous update, G1 stable state attracting most trajectories	GINsim (adapted model)
Fauré et al., 2006	Mammals	Boolean model; Regulatory graph and standard logical functions (10)	Synchronous, asynchronous and mixed updating scheme; cyclic attractor plus quiescent stable state	GINsim Cell Collective
Davidich and Bornholdt, 2008	Fission Yeast	Boolean model; Threshold logical functions (10)	Synchronous update, G1 stable state attracting most trajectories	GINsim (adapted model)
Irons, 2009	Budding Yeast	Boolean model; Regulatory graph with 4 phenomenological nodes and standard logical functions (18)	Synchronous and temporized updating schemes, single cyclic attractor	GINsim Cell Collective
Fauré et al., 2009	Budding Yeast	Multilevel model; Regulatory graph and standard logical functions (32)	Priority classes, single cyclic attractor	GINsim
Sahin et al., 2009	Human	Boolean model; Regulatory graph and standard logical functions (20)	Asynchronous update, 3 stable states, transient oscillations	GINsim Cell Collective
Todd and Helikar, 2012	Budding Yeast	Boolean model; based on Irons (2009)'s model (20)	Analysis over variation of inputs, which are allocated probaiblities to be active	Cell Collective
Flobak et al., 2015	Human	Boolean model; Regulatory graph and standard logical functions, no input (77)	Asynchronous update on a reduced model, a single stable state denoting cell proliferation	GINsim

The studies listed in Table 2 rely on different modeling assumptions (e.g., using generic or specific rules, and considering specific updating schemes). By and large, relying on qualitative information, the authors were able to capture the succession of key events involved in cell cycle. Moreover, several studies recapitulate the effect of various kinds of perturbations (losses- or gains-of-function, see e.g., Fauré et al., 2006; Fauré et al., 2009; Irons, 2009). Fauré and Thieffry (2009) published a comparative review of cell cycle logical models (predating 2009). An interesting observation was the conservation of a functional negative regulatory circuit at the core of the cell cycle engine, involving cyclin B and Cdc20 (or their orthologs in other species), as well as of several coupled positive regulatory circuits. Here, we restrict ourselves to a few studies in order to emphasize specific aspects of logical modeling analyses.

Based on the differential model proposed by Novák and Tyson (2004) and Fauré et al. (2006) defined a Boolean model for the core network driving the entry of mammalian cells into cell cycle. This model accounts for the existence of a quiescent stable state (in the absence of growth factors, represented by the shutoff of cyclin D, the input component), as well as for a cyclic attractor characterized by the periodic activities of the cyclins A, B and E, which drive the cell cycle through key transitions by enabling the phosphorylation of a number of substrates by their catalytic partners, the cyclin-dependent kinases (CDKs). This model further includes the three main inhibitors of the cell cycle: the retinoblastoma protein Rb, the CDK inhibitor p27/Kip1 and the proteasome complex represented by its two co-activators Cdh1 and Cdc20. Finally, this model accounts for the role of the E2 ubiquitin conjugating enzyme UbcH10, which participates in Cdh1 dependent degradation of cyclin A. This extension of the original differential model explains how the auto-ubiquitination of UbcH10 probably prevents cyclin A from degradation by the APC in G1 phase. Complex formation and protein sequestration were modeled in terms of logical rules associated with the target proteins, which enabled the author to keep the number of components considered to the low end (ten components). Although very simplified, this model broadly reproduced the sequence of molecular events along the normal cell cycle, for both synchronous and asynchronous updating schemes. The authors further considered a list of documented perturbations to validate their model. Although the simulations of various perturbations were shown to match experimental observations, it was not the case for some documented perturbations, including for a knock-out of cyclin E.

**Figure 7:**
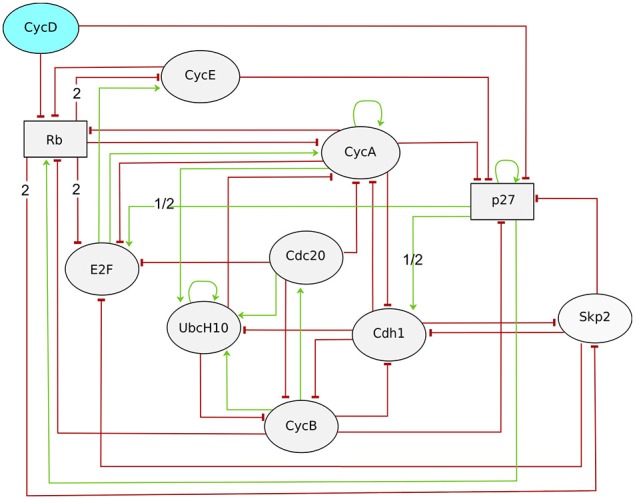
**Regulatory graph of the mammalian cell cycle model (Traynard et al., 2015)**. The input node, CycD accounts for the positive signal, as Cyclin D is activated by growth factors. All components are Boolean, except Rb and p27 (see Text). Interactions requiring the higher threshold (value 2) or having different effect depending on the threshold value (1/2) are labeled accordingly.

Traynard et al. (2015) revisited this model to solve the remaining discrepancies in the light of recent data (see Figure 7). As hinted already in the seminal study by Fauré et al. (2006), the authors considered the use of a ternary variable for the cell cycle inhibitor Rb, which can be phosphorylated at multiple sites, associated with different activities. Similarly, they associated a ternary variable with p27 to account for its significant but incomplete degradation in the presence of CycD and in the absence of CycA and CycE. They further included the F-box protein Skp2 in the model. Skp2 promotes the degradation of phosphorylated p27 and thereby enables its degradation. Skp2 degradation is promoted by Rb binding to Cdh1. Skp2 thus links the two cell cycle repressors Rb and p27, and provides an additional mechanism by which Rb can arrest the cell cycle. In order to assess the benefits of each modification, model checking was used to verify the existence (or the absence) of specific trajectories characteristic of the cell cycle dynamics. More specifically, a generic CTL temporal logical formula (see Section 3.1) was used to verify the existence of a trajectory complying with a sequence *S*_1_, *S*_2_, *S*_3_, …, *S*_*n*−1_, *S*_*n*_, each denoting a set of states defined by constraints on some of the model components:

$$\begin{array}{l} \text{INIT }S_{1}\text{; SPEC !E(}S_{1})\text{ U }(S_{2}\&\text{E}[(S_{2})\text{ U}\\ \;(S_{3}\&...\text{E}[(S_{\text{n}-1})\text{ U }(S_{\text{n}})])])]. \end{array}$$

Here, the negation (denoted by the operator !) is used to obtain a counter-example, from the model checker, whenever the property is false, containing the desired trajectory complying with a sequence *S*_1_, *S*_2_, *S*_3_, …, *S*_*n*−1_, *S*_*n*_. As a result, the authors obtained a generic multi-valued logical model of the mammalian cell cycle that qualitatively matches the most salient dynamical properties of the normal cell cycle, in particular at the G1/S transition, as well as the phenotypes of many mutants (Traynard et al., 2015).

**Figure 8:**
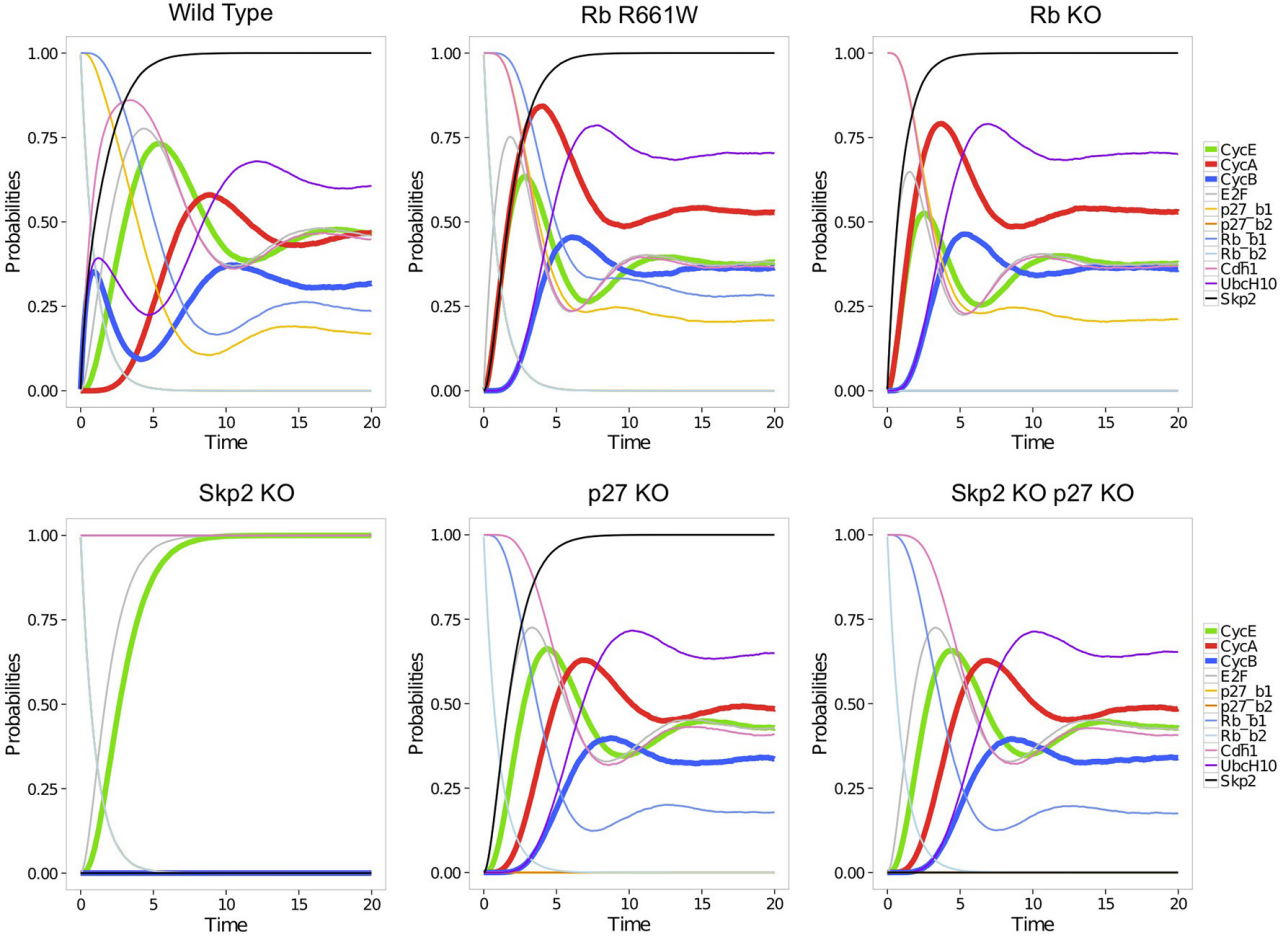
**Stochastic trajectories simulated with MaBoSS for each component of the model of Figure 7, with equal rates for all transitions**. From top left to bottom right: simulations without perturbation (wild-type); with a perturbation corresponding to the partial mutation RbR661W annihilating the repressing activity of Rb on E2F; Rb loss-of-function; Skp2 loss-of-function; p27 loss-of-function; combination of Skp2 and p27 loss-of-functions (Traynard et al., 2015). Rb_b1 and Rb_b2 are the two Boolean variables used to represent the levels of Rb (0,1, and 2). Similarly, p27_b1 and p27_b2 account for the levels of p27.

More quantitative characterizations of asymptotic behaviors can be provided by stochastic simulations using MaBoSS (see Section 2.3). As MaBoSS is restricted to Boolean models, the ternary node Rb was split into two Boolean nodes Rb1 and Rb2, associated with the first and second Rb thresholds, respectively (and similarly for p27). The stochastic trajectories computed for this model reflect the kinetics of the cell cycle progression driven by the input cyclin D (see Figure 8). Transient oscillations can be observed as the trajectories all start in G0 (with Rb1, Rb2, p27, and Cdh1 the only active nodes) and progressively desynchronize. It is particularly interesting to compare the trajectories obtained for wild type (WT) vs. perturbed conditions. The trajectories obtained for five perturbations illustrate the role of Rb and of the pathway Rb-Skp2-p27 in the model (Figure 8). Two perturbations were considered for Rb: the full loss-of-function (Rb KO), and a partial loss-of-function, where Rb loses its ability to repress E2F, but conserves its repressing activity on Skp2 (Rb R661W). The resulting stochastic trajectories highlight the role of Rb in the sequential activation of cyclin E and cyclin A, ensured by the repressing activity of the two underphosphorylated forms of Rb on E2F: in the WT case, the activation of cyclin A is clearly delayed relatively to the activation of cyclin E. In contrast, in the absence of the repressing effect of Rb on E2F, cyclin E and cyclin A are activated at the same time. The lack of significant difference between the trajectories of Rb R661W and Rb KO suggests that the repression of Skp2 by Rb has no major impact on the cell cycle. However, this interaction is necessary to ensure the quiescent state in the absence of cyclin D. Skp2 loss-of-function (Skp2 KO) arrests the cell cycle (Figure 8), presumably due to the stabilization of p27. Indeed, the oscillations are restored in the double mutant Skp2 KO p27 KO.

In an independent study focusing on Yeast cell cycle control, Todd and Helikar (2012) built on the model of Irons (2009) and showed that cell phenotypes can be modeled as ergodic sets irreducible sets of states of the corresponding Markov chain; i.e., set of states that cannot be left once reached), by defining probabilities for the input components to be active and modeling these signals as continuous variables. In this work, the cell cycle was analyzed as a sequence of models, each accounting for a specific phase of the cycle, which allowed to characterize the (continuous) dynamics of all regulatory components along each phase, and more closely compare them to various experimental observations. Modeling extracellular signals as continuous variables (i.e., cell size) resulted in the finding that the yeast cell cycle network is stable under different patterns of cell growth. That is, as long as the checkpoints are appropriately activated (i.e., the environment is stable enough for the successful completion of the current phase), the modeled cell progresses through the cycle, independently of its size. Furthermore, the continuous dynamics of the model components were found consistent with various experimental studies.

## 6. Discussion

After introducing the logical modeling framework and a range of methodological advances to analyze dynamical properties of these discrete models, we have presented a number of assets of this approach through two important case studies. Here, we discuss further issues and complementary approaches.

Besides the consideration of probabilistic input values, we have focused on non-stochastic models (recall that asynchronous dynamics is non-deterministic but not random). However, several methods have been proposed to include noise in Boolean models. For example, accounting for uncertainty in the regulatory functions, Shmulevich et al. (2002) associate each component with a set of regulatory functions, one being randomly selected at each step of the simulation. Another option consists in randomly taking the complements of the regulatory function outcomes (Alvarez-Buylla et al., 2008). In Garg et al. (2009), the authors consider potential failures of the regulatory functions. For all these stochastic variants, the synchronous scheme was adopted.

It is worth mentioning that several continuous transpositions of logical models have been proposed, for example considering fuzzy logic (Aldridge et al., 2009; Morris et al., 2011), or transforming Boolean models into ordinary differential equations (Mendoza and Xenarios, 2006; Wittmann et al., 2009). The reverse transformation has been formally addressed for the specific class of piecewise affine differential models (Batt et al., 2008; Chaves et al., 2010).

As shown in Section 3 with the usage of model checking, logical models are amenable to sophisticated formal methods. Initially developed for software and hardware systems, these techniques are indeed well adapted for logical model identification (e.g., constraint programming, see Corblin et al., 2010 and Answer Set Programming, see Videla et al., 2015) and for model analysis (e.g., satisfiability problem (SAT) for the identification of the attractors of Boolean models, see Dubrova and Teslenko, 2011).

Although progress has been made with the definition of SBML qual, the SBML Level 3 Qualitative Models Package (Chaouiya et al., 2013), further efforts are needed to ensure model exchange, reuse and extension. A first issue concerns reproducibility of modeling studies. This can be achieved first by providing model files, in BioModels database (Chelliah et al., 2015), or in model repositories such as those provided by Cell Collective or GINsim (see Section 2.3). Second, modeling assumptions and simulation settings should be precisely described. For example, we have underlined that model properties can vary depending on the adopted updating scheme (Section 2.2). Furthermore, model extensions often simply refer to the addition of components, but it can also consists in refining the model with a stochastic extension (e.g., with probabilistic input values as in Figure 6). The different formalism extensions evoked above together with many others still need to be precisely characterized, managed within a control vocabulary and supported in a future SBML qual version. Further integration with core SBML Level 3 concepts will be needed to support the encoding of hybrid models combining features of both discrete and continuous formalisms. It is the purpose of CoLoMoTo (the Consortium for Logical Models and Tools) to stimulate and coordinate such developments.

## Author contributions

WA, PT, and PM equally contributed to the manuscript, which content and organization have been designed by CC, DT, TH, and JS. CC coordinated the writing of the manuscript, and contributed particularly to Sections 2, 3. PM contributed to Section 3 in particular. JSR contributed to Section 4 and revised the manuscript. DT supervised the writing of Section 5 and particularly contributed to Sections 1, 5, and 6. WA contributed to Section 5.1 and PT to Section 5.2. TH contributed to Sections 4, 5. All authors have read and approved the final manuscript.

## Funding

WA has been supported by postdoctoral grants from the LabEx MemoLife and from the Ecole Normale Supérieure. ENS team further acknowledges the support of the French *Plan Cancer* (2014–2017), in the context of the project entitled *Modeling cell communication networks in breast cancer - CoMET*. PM was supported by FCT grants UID/CEC/50021/2013 and IF/01333/2013. CC acknowledges the support of the Fundação Calouste Gulbenkian.

### Conflict of interest statement

The authors declare that the research was conducted in the absence of any commercial or financial relationships that could be construed as a potential conflict of interest.

## Supplementary material

Supplementary Figure S1 **This figure provides an illustration of a simple multi-valued model and its asynchronous dynamics represented as a State Transition Graph and as a Hierarchical Transition Graph**.

## References

[B1] Abou-Jaoudé W. Monteiro P. T. Naldi A. Grandclaudon M. Soumelis V. Chaouiya C.. (2015). Model checking to assess T-helper cell plasticity. Front. Bioeng. Biotechnol. 2(Suppl. 1):86. 10.3389/fbioe.2014.00086 25674559 PMC4309205

[B2] Albert R. Thakar J. (2014). Boolean modeling: a logic-based dynamic approach for understanding signaling and regulatory networks and for making useful predictions. Wiley Interdisc. Rev. Syst. Biol. Med. 6, 353–369. 10.1002/wsbm.1273 25269159

[B3] Aldridge B. B. Saez-Rodriguez J. Muhlich J. L. Sorger P. K. Lauffenburger D. A. (2009). Fuzzy logic analysis of kinase pathway crosstalk in tnf/egf/insulin-induced signaling. PLoS Comput. Biol. 5:e1000340. 10.1371/journal.pcbi.1000340 19343194 PMC2663056

[B4] Alvarez-Buylla E. R. Chaos A. Aldana M. Benítez M. Cortes-Poza Y. Espinosa-Soto C.. (2008). Floral morphogenesis: stochastic explorations of a gene network epigenetic landscape. PLoS ONE 3:e3626. 10.1371/journal.pone.0003626 18978941 PMC2572848

[B5] Antebi Y. E. Reich-Zeliger S. Hart Y. Mayo A. Eizenberg I. Rimer J.. (2013). Mapping differentiation under mixed culture conditions reveals a tunable continuum of T cell fates. PLoS Biol. 11:e1001616. 10.1371/journal.pbio.1001616 23935451 PMC3728017

[B6] Arellano G. Argil J. Azpeitia E. Benítez M. Carrillo M. Góngora P.. (2011). “Antelope”: a hybrid-logic model checker for branching-time Boolean GRN analysis. BMC Bioinformatics 12:490. 10.1186/1471-2105-12-490 22192526 PMC3316443

[B7] Batt G. de Jong H. Page M. Geiselmann J. (2008). Symbolic reachability analysis of genetic regulatory networks using discrete abstractions. Automatica 44, 982–989. 10.1016/j.automatica.2007.08.004

[B8] Batt G. Ropers D. de Jong H. Geiselmann J. Mateescu R. Page M.. (2005). Validation of qualitative models of genetic regulatory networks by model checking: analysis of the nutritional stress response in Escherichia coli. Bioinformatics 21(Suppl. 1):i19–i28. 10.1093/bioinformatics/bti1048 15961457

[B9] Bérenguier D. Chaouiya C. Monteiro P. T. Naldi A. Remy E. Thieffry D.. (2013). Dynamical modeling and analysis of large cellular regulatory networks. Chaos 23:025114. 10.1063/1.4809783 23822512

[B10] Bonzanni N. Garg A. Feenstra K. A. Schütte J. Kinston S. Miranda-Saavedra D.. (2013). Hard-wired heterogeneity in blood stem cells revealed using a dynamic regulatory network model. Bioinformatics 29, i80–i88. 10.1093/bioinformatics/btt243 23813012 PMC3694641

[B11] Bornholdt S. (2008). Boolean network models of cellular regulation: prospects and limitations. J. R. Soc. Interface 5(Suppl. 1), S85–S94. 10.1098/rsif.2008.0132.focus 18508746 PMC2386560

[B12] Brim L. Češka M. Šafránek D. (2013). Model checking of biological systems, in Formal Methods for Dynamical Systems, Volume 7938 of em Lecture Notes in Computer Science, eds Bernardo M. Vink E. Pierro A. Wiklicky H. (Bertinoro: Springer), 63–112.

[B13] Chabrier N. Fages F. (2003). Symbolic model checking of biochemical networks, in Computational Methods in Systems Biology, Volume 2602 of Lecture Notes in Computer Science, ed Priami C. (Rovereto: Springer), 149–162. 10.1007/3-540-36481-1_13

[B14] Chaouiya C. Bérenguier D. Keating S. M. Naldi A. van Iersel M. P. Rodriguez N.. (2013). SBML qualitative models: a model representation format and infrastructure to foster interactions between qualitative modelling formalisms and tools. BMC Syst. Biol. 7:135. 10.1186/1752-0509-7-135 24321545 PMC3892043

[B15] Chaouiya C. Keating S. M. Berenguier D. Naldi A. Thieffry D. van Iersel M. P.. (2015). The systems biology markup language (SBML) level 3 package: qualitative models, version 1, release 1. J. Integr. Bioinform. 12:270. 10.2390/biecoll-jib-2015-270 26528568

[B16] Chaouiya C. Naldi A. Thieffry D. (2012). Logical modelling of gene regulatory networks with ginsim. Methods Mol. Biol. 804, 463–479. 10.1007/978-1-61779-361-5_23 22144167

[B17] Chaves M. Albert R. Sontag E. D. (2005). Robustness and fragility of Boolean models for genetic regulatory networks. J. Theor. Biol. 235, 431–449. 10.1016/j.jtbi.2005.01.023 15882705

[B18] Chaves M. Tournier L. Gouzé J.-L. (2010). Comparing Boolean and piecewise affine differential models for genetic networks. Acta Biotheoretica 58, 217–232. 10.1007/s10441-010-9097-6 20665073

[B19] Chelliah V. Juty N. Ajmera I. Ali R. Dumousseau M. Glont M.. (2015). Biomodels: ten-year anniversary. Nucl. Acids Res. 43(Database issue):D542–D548. 10.1093/nar/gku1181 25414348 PMC4383975

[B20] Cimatti A. Clarke E. M. Giunchiglia E. Giunchiglia F. Pistore M. Roveri M. (2002). NuSMV2: an OpenSource tool for symbolic model checking, in Computer Aided Verification, Volume 2404 of Lecture Notes in Computer Science, eds Brinksma E. Larsen K. G. (Copenhagen: Springer), 359–364.

[B21] Clarke E. M. Faeder J. R. Langmead C. J. Harris L. A. Jha S. K. Legay A. (2008). Statistical model checking in BioLab: applications to the automated analysis of T-cell receptor signaling pathway, in Computational Methods in Systems Biology, Volume 5307 of Lecture Notes in Bioinformatics, eds Heiner M. Uhrmacher A. M. (Rostock: Springer), 231–250. 10.1007/978-3-540-88562-7_18

[B22] Clarke E. M. Grumberg O. Peled D. A. (1999). Model Checking. Cambridge, MA: MIT Press.

[B23] Cohen D. P. A. Martignetti L. Robine S. Barillot E. Zinovyev A. Calzone L. (2015). Mathematical modelling of molecular pathways enabling tumour cell invasion and migration. PLoS Comput. Biol. 11:e1004571. 10.1371/journal.pcbi.1004571 26528548 PMC4631357

[B24] Conroy B. D. Herek T. A. Shew T. D. Latner M. Larson J. J. Allen L.. (2014). Design, assessment, and *in vivo* evaluation of a computational model illustrating the role of CAV1 in CD4(+) T-lymphocytes. Front. Immunol. 5:599. 10.3389/fimmu.2014.00599 25538703 PMC4257089

[B25] Corblin F. Fanchon E. Trilling L. (2010). Applications of a formal approach to decipher discrete genetic networks. BMC Bioinformatics 11:385. 10.1186/1471-2105-11-385 20646302 PMC2918581

[B26] Crespo I. Krishna A. Le Béchec A. del Sol A. (2013). Predicting missing expression values in gene regulatory networks using a discrete logic modeling optimization guided by network stable states. Nucl. Acids Res. 41:e8. 10.1093/nar/gks785 22941654 PMC3592407

[B27] Davidich M. Bornholdt S. (2008). The transition from differential equations to Boolean networks: a case study in simplifying a regulatory network model. J. Theor. Biol. 255, 269–277. 10.1016/j.jtbi.2008.07.020 18692073

[B28] Dubrova E. Teslenko M. (2011). A SAT-based algorithm for finding attractors in synchronous boolean networks. IEEE/ACM Trans. Comput. Biol. Bioinform. 8, 1393–1399. 10.1109/tcbb.2010.20 21778527

[B29] Fauré A. Naldi A. Chaouiya C. Thieffry D. (2006). Dynamical analysis of a generic boolean model for the control of the mammalian cell cycle. Bioinformatics 22, e124–e131. 10.1093/bioinformatics/btl210 16873462

[B30] Fauré A. Naldi A. Lopez F. Chaouiya C. Ciliberto A. Thieffry D. (2009). Modular logical modelling of the budding yeast cell cycle. Mol. BioSyst. 5, 1787–1796. 10.1039/b910101m 19763337

[B31] Fauré A. Thieffry D. (2009). Logical modelling of cell cycle control in eukaryotes: a comparative study. Mol. Biosyst. 5, 1569–1581. 10.1039/b907562n 19763341

[B32] Fauré A. Vreede B. M. I. Sucena E. Chaouiya C. (2014). A discrete model of drosophila eggshell patterning reveals cell-autonomous and juxtacrine effects. PLoS Comput. Biol. 10:e1003527. 10.1371/journal.pcbi.1003527 24675973 PMC3967936

[B33] Ferrell J. E. Jr. Tsai T. Y.-C. Yang Q. (2011). Modeling the cell cycle: why do certain circuits oscillate? Cell 144, 874–885. 10.1016/j.cell.2011.03.006 21414480

[B34] Flobak Å. Baudot A. Remy E. Thommesen L. Thieffry D. Kuiper M.. (2015). Discovery of drug synergies in gastric cancer cells predicted by logical modeling. PLoS Comput. Biol. 11:e1004426. 10.1371/journal.pcbi.1004426 26317215 PMC4567168

[B35] Garg A. Di Cara A. Xenarios I. Mendoza L. De Micheli G. (2008). Synchronous versus asynchronous modeling of gene regulatory networks. Bioinformatics 24, 1917–1925. 10.1093/bioinformatics/btn336 18614585 PMC2519162

[B36] Garg A. Mohanram K. Di Cara A. De Micheli G. Xenarios I. (2009). Modeling stochasticity and robustness in gene regulatory networks. Bioinformatics 25, i101–i109. 10.1093/bioinformatics/btp214 19477975 PMC2687968

[B37] Glass L. Kauffman S. A. (1973). The logical analysis of continuous, non-linear biochemical control networks. J. Theor. Biol. 39, 103–129. 10.1016/0022-5193(73)90208-7 4741704

[B38] González A. Chaouiya C. Thieffry D. (2008). Logical modelling of the role of the hh pathway in the patterning of the drosophila wing disc. Bioinformatics 24, i234–i240. 10.1093/bioinformatics/btn266 18689831

[B39] Grieco L. Calzone L. Bernard-Pierrot I. Radvanyi F. Kahn-Perlès B. Thieffry D. (2013). Integrative modelling of the influence of MAPK network on cancer cell fate decision. PLoS Comput. Biol. 9:e1003286. 10.1371/journal.pcbi.1003286 24250280 PMC3821540

[B40] Helikar T. Kochi N. Kowal B. Dimri M. Naramura M. Raja S. M.. (2013a). A comprehensive, multi-scale dynamical model of ErbB receptor signal transduction in human mammary epithelial cells. PLoS ONE 8:e61757. 10.1371/journal.pone.0061757 23637902 PMC3630219

[B41] Helikar T. Kowal B. McClenathan S. Bruckner M. Rowley T. Madrahimov A.. (2012). The Cell Collective: toward an open and collaborative approach to systems biology. BMC Syst. Biol. 6:96. 10.1186/1752-0509-6-96 22871178 PMC3443426

[B42] Helikar T. Kowal B. Rogers J. A. (2013b). A cell simulator platform: the cell collective. Clin. Pharmacol. Ther. 93, 393–395. 10.1038/clpt.2013.41 23549147 PMC5242230

[B43] Helikar T. Rogers J. A. (2009). Chemchains: a platform for simulation and analysis of biochemical networks aimed to laboratory scientists. BMC Syst. Biol. 3:58. 10.1186/1752-0509-3-58 19500393 PMC2705353

[B44] Hinton A. Kwiatkowska M. Norman G. Parker D. (2006). PRISM: a tool for automatic verification of probabilistic systems, in Tools and Algorithms for the Construction and Analysis of Systems, Volume 3920 of Lecture Notes in Computer Science, eds Hermanns H. Palsberg J. (Vienna: Springer), 441–444. 10.1007/11691372_29

[B45] Huang S. Ernberg I. Kauffman S. (2009). Cancer attractors: a systems view of tumors from a gene network dynamics and developmental perspective. Semin. Cell Dev. Biol. 20, 869–876. 10.1016/j.semcdb.2009.07.003 19595782 PMC2754594

[B46] Hucka M. Finney A. Sauro H. M. Bolouri H. Doyle J. C. Kitano H.. (2003). The systems biology markup language (SBML): a medium for representation and exchange of biochemical network models. Bioinformatics 19, 524–531. 10.1093/bioinformatics/btg015 12611808

[B47] Irons D. J. (2009). Logical analysis of the budding yeast cell cycle. J. Theor. Biol. 257, 543–559. 10.1016/j.jtbi.2008.12.028 19185585

[B48] Jacob F. Monod J. (1961). Genetic regulatory mechanisms in the synthesis of proteins. J. Mol. Biol. 3, 318–356. 10.1016/s0022-2836(61)80072-7 13718526

[B49] Kauffman S. A. (1969). Metabolic stability and epigenesis in randomly constructed genetic nets. J. Theor. Biol. 22, 437–467. 10.1016/0022-5193(69)90015-0 5803332

[B50] Kauffman S. A. (1993). The Origins of Order: Self-Organization and Selection in Evolution. New York, NY: Oxford University Press.

[B51] Keller R. Klein M. Thomas M. Dräger A. Metzger U. Templin M. F.. (2016). Coordinating role of RXRα in downregulating hepatic detoxification during inflammation revealed by fuzzy-logic modeling. PLoS Comput. Biol. 12:e1004431. 10.1371/journal.pcbi.1004431 26727233 PMC4699813

[B52] Kirouac D. C. Du J. Y. Lahdenranta J. Overland R. Yarar D. Paragas V.. (2013). Computational modeling of ERBB2-amplified breast cancer identifies combined ErbB2/3 blockade as superior to the combination of MEK and AKT inhibitors. Sci. Signal. 6:ra68. 10.1126/scisignal.2004008 23943608

[B53] Klamt S. Haus U.-U. Theis F. (2009). Hypergraphs and cellular networks. PLoS Comput. Biol. 5:e1000385. 10.1371/journal.pcbi.1000385 19478865 PMC2673028

[B54] Klarner H. Bockmayr A. Siebert H. (2015). Computing maximal and minimal trap spaces of Boolean networks. Nat. Comput. 14, 535–544. 10.1007/s11047-015-9520-7

[B55] Le Novère N. (2015). Quantitative and logic modelling of molecular and gene networks. Nat. Rev. Genet. 16, 146–158. 10.1038/nrg3885 25645874 PMC4604653

[B56] Le Novère N. Finney A. Hucka M. Bhalla U. S. Campagne F. Collado-Vides J.. (2005). Minimum information requested in the annotation of biochemical models (MIRIAM). Nat. Biotechnol. 23, 1509–1515. 16333295 10.1038/nbt1156

[B57] Li F. Long T. Lu Y. Ouyang Q. Tang C. (2004). The yeast cell-cycle network is robustly designed. Proc. Natl. Acad. Sci. U.S.A. 101, 4781–4786. 10.1073/pnas.0305937101 15037758 PMC387325

[B58] Lomuscio A. Pecheur C. Raimondi F. (2007). Automatic verification of knowledge and time with NuSMV, in International Joint Conference on Artificial Intelligence, ed Veloso M. M. (Hyderabad: AAAI), 1384–1389.

[B59] MacNamara A. Terfve C. Henriques D. Bernabé B. P. Saez-Rodriguez J. (2012). State-time spectrum of signal transduction logic models. Phys. Biol. 9:045003. 10.1088/1478-3975/9/4/045003 22871648

[B60] Madrahimov A. Helikar T. Kowal B. Lu G. Rogers J. (2013). Dynamics of influenza virus and human host interactions during infection and replication cycle. Bull. Math. Biol. 75, 988–1011. 10.1007/s11538-012-9777-2 23081726

[B61] Martinez-Sanchez M. E. Mendoza L. Villarreal C. Álvarez-Buylla E. R. (2015). A minimal regulatory network of extrinsic and intrinsic factors recovers observed patterns of CD4+ T cell differentiation and plasticity. PLoS Comput. Biol. 11:e1004324. 10.1371/journal.pcbi.1004324 26090929 PMC4475012

[B62] Martínez-Sosa P. Mendoza L. (2013). The regulatory network that controls the differentiation of T lymphocytes. BioSystems 113, 96–103. 10.1016/j.biosystems.2013.05.007 23743337

[B63] Mendes N. D. Monteiro P. T. Carneiro J. Remy E. Chaouiya C. (2014). Quantification of reachable attractors in asynchronous discrete dynamics. arXiv:1411.3539 [cs.DM].

[B64] Mendoza L. (2006). A network model for the control of the differentiation process in Th cells. BioSystems 84, 101–114. 10.1016/j.biosystems.2005.10.004 16386358

[B65] Mendoza L. Xenarios I. (2006). A method for the generation of standardized qualitative dynamical systems of regulatory networks. Theor. Biol. Med. Model. 3:13. 10.1186/1742-4682-3-13 16542429 PMC1440308

[B66] Miskov-Zivanov N. Turner M. S. Kane L. P. Morel P. A. Faeder J. R. (2013). The duration of T cell stimulation is a critical determinant of cell fate and plasticity. Sci. Signal. 6:ra97. 10.1126/scisignal.2004217 24194584 PMC4074924

[B67] Mombach J. C. M. Bugs C. A. Chaouiya C. (2014). Modelling the onset of senescence at the G1/S cell cycle checkpoint. BMC Genomics 15(Suppl. 7):S7. 10.1186/1471-2164-15-S7-S7 25573782 PMC4243082

[B68] Monod J. Jacob F. (1961). Teleonomic mechanisms in cellular metabolism, growth, and differentiation. Cold Spring Harb. Symp. Quan. Biol. 26, 389–401. 10.1101/SQB.1961.026.01.048 14475415

[B69] Monteiro P. T. Chaouiya C. (2012). Efficient verification for logical models of regulatory networks, in PACBB, Volume 154 of Advances in Intelligent and Soft Computing, eds Rocha M. P. Luscombe N. Fdez-Riverola F. Corchado Rodríguez J. M. (Berlin; Heidelberg: Springer), 259–267.

[B70] Morris M. K. Saez-Rodriguez J. Clarke D. C. Sorger P. K. Lauffenburger D. A. (2011). Training signaling pathway maps to biochemical data with constrained fuzzy logic: quantitative analysis of liver cell responses to inflammatory stimuli. PLoS Comput. Biol. 7:e1001099. 10.1371/journal.pcbi.1001099 21408212 PMC3048376

[B71] Morris M. K. Saez-Rodriguez J. Sorger P. K. Lauffenburger D. A. (2010). Logic-based models for the analysis of cell signaling networks. Biochemistry 49, 3216–3224. 10.1021/bi902202q 20225868 PMC2853906

[B72] Murphy K. Travers P. Walport M. Janeway C. (2012). Janeway's Immunology. New York, NY: Garland Science.

[B73] Nakayamada S. Takahashi H. Kanno Y. O'shea J. J. (2012). Helper T cell diversity and plasticity. Curr. Opin. Immunol. 24, 297–302. 10.1016/j.coi.2012.01.014 22341735 PMC3383341

[B74] Naldi A. Carneiro J. Chaouiya C. Thieffry D. (2010). Diversity and plasticity of Th cell types predicted from regulatory network modelling. PLoS Comput. Biol. 6:e1000912. 10.1371/journal.pcbi.1000912 20824124 PMC2932677

[B75] Naldi A. Monteiro P. T. Chaouiya C. (2012). Efficient handling of large signalling-regulatory networks by focusing on their core control, in Computational Methods in Systems Biology, Volume 7605 of Lecture Notes in Computer Science, eds Gilbert D. Heiner M. (London: Springer), 288–306. 10.1007/978-3-642-33636-2_17

[B76] Naldi A. Monteiro P. T. Müssel C. Consortium for Logical Models Tools Kestler, H. A. Thieffry D.. (2015). Cooperative development of logical modelling standards and tools with CoLoMoTo. Bioinformatics 31, 1154–1159. 10.1093/bioinformatics/btv013 25619997

[B77] Naldi A. Remy E. Thieffry D. Chaouiya C. (2011). Dynamically consistent reduction of logical regulatory graphs. Theor. Comput. Sci. 412, 2207–2218. 10.1016/j.tcs.2010.10.021

[B78] Naldi A. Thieffry D. Chaouiya C. (2007). Decision diagrams for the representation and analysis of logical models of genetic networks, in Computational Methods in Systems Biology, Volume 4695 of Lecture Notes in Computer Science, eds Calder M. Gilmore S. (Edinburgh: Springer), 233–247. 10.1007/978-3-540-75140-3_16

[B79] Novák B. Tyson J. J. (2004). A model for restriction point control of the mammalian cell cycle. J. Theor. Biol. 230, 563–579. 10.1016/j.jtbi.2004.04.039 15363676

[B80] Oyeyemi O. J. Davies O. Robertson D. L. Schwartz J.-M. (2015). A logical model of HIV-1 interactions with the T-cell activation signalling pathway. Bioinformatics 31, 1075–1083. 10.1093/bioinformatics/btu787 25431332

[B81] Puniya B. L. Allen L. Hochfelder C. Majumder M. Helikar T. (2016). Systems perturbation analysis of a large scale signal transduction model reveals potentially influential candidates for cancer therapeutics. Front. Bioeng. Biotechnol. 4:10. 10.3389/fbioe.2016.00010 26904540 PMC4750464

[B82] Remy E. Mossé B. Chaouiya C. Thieffry D. (2003). A description of dynamical graphs associated to elementary regulatory circuits. Bioinformatics 19(Suppl. 2), ii172–ii178. 10.1093/bioinformatics/btg1075 14534187

[B83] Remy E. Rebouissou S. Chaouiya C. Zinovyev A. Radvanyi F. Calzone L. (2015). A modeling approach to explain mutually exclusive and co-occurring genetic alterations in bladder tumorigenesis. Cancer Res. 75, 4042–4052. 10.1158/0008-5472.CAN-15-0602 26238783

[B84] Saadatpour A. Albert R. Reluga T. C. (2013). A reduction method for Boolean network models proven to conserve attractors. SIAM J. Appl. Dyn. Syst. 12, 1997–2011. 10.1137/13090537x PMC7597850 33132767

[B85] Saadatpour A. Wang R.-S. Liao A. Liu X. Loughran T. P. Albert I.. (2011). Dynamical and structural analysis of a T cell survival network identifies novel candidate therapeutic targets for large granular lymphocyte leukemia. PLoS Comput. Biol. 7:e1002267. 10.1371/journal.pcbi.1002267 22102804 PMC3213185

[B86] Saez-Rodriguez J. Alexopoulos L. G. Epperlein J. Samaga R. Lauffenburger D. A. Klamt S.. (2009). Discrete logic modelling as a means to link protein signalling networks with functional analysis of mammalian signal transduction. Mol. Syst. Biol. 5:331. 10.1038/msb.2009.87 19953085 PMC2824489

[B87] Saez-Rodriguez J. Simeoni L. Lindquist J. A. Hemenway R. Bommhardt U. Arndt B.. (2007). A logical model provides insights into T cell receptor signaling. PLoS Comput. Biol. 3:e163. 10.1371/journal.pcbi.0030163 17722974 PMC1950951

[B88] Sahin O. Fröhlich H. Löbke C. Korf U. Burmester S. Majety M.. (2009). Modeling ERBB receptor-regulated G1/S transition to find novel targets for de novo trastuzumab resistance. BMC Syst. Biol. 3:1. 10.1186/1752-0509-3-1 19118495 PMC2652436

[B89] Samaga R. Klamt S. (2013). Modeling approaches for qualitative and semi-quantitative analysis of cellular signaling networks. Cell Commun. Signal 11:43. 10.1186/1478-811X-11-43 23803171 PMC3698152

[B90] Sánchez L. Chaouiya C. Thieffry D. (2008). Segmenting the fly embryo: logical analysis of the role of the segment polarity cross-regulatory module. Int. J. Dev. Biol. 52, 1059–1075. 10.1387/ijdb.072439ls 18956339

[B91] Schlatter R. Schmich K. Avalos Vizcarra I. Scheurich P. Sauter T. Borner C.. (2009). ON/OFF and beyond–a Boolean model of apoptosis. PLoS Comput. Biol. 5:e1000595. 10.1371/journal.pcbi.1000595 20011108 PMC2781112

[B92] Shmulevich I. Dougherty E. R. Kim S. Zhang W. (2002). Probabilistic boolean networks: a rule-based uncertainty model for gene regulatory networks. Bioinformatics 18, 261–274. 10.1093/bioinformatics/18.2.261 11847074

[B93] Stoll G. Viara E. Barillot E. Calzone L. (2012). Continuous time Boolean modeling for biological signaling: application of Gillespie algorithm. BMC Syst. Biol. 6:116. 10.1186/1752-0509-6-116 22932419 PMC3517402

[B94] Sugita M. (1963). Functional analysis of chemical systems *in vivo* using a logical circuit equivalent. II. The idea of a molecular automation. J. Theor. Biol. 4, 179–192. 10.1016/0022-5193(63)90087-0 5875160

[B95] Terfve C. Cokelaer T. Henriques D. MacNamara A. Goncalves E. Morris M. K.. (2012). CellNOptR: a flexible toolkit to train protein signaling networks to data using multiple logic formalisms. BMC Syst. Biol. 6:133. 10.1186/1752-0509-6-133 23079107 PMC3605281

[B96] Terfve C. D. A. Wilkes E. H. Casado P. Cutillas P. R. Saez-Rodriguez J. (2015). Large-scale models of signal propagation in human cells derived from discovery phosphoproteomic data. Nat. Commun. 6:8033. 10.1038/ncomms9033 26354681 PMC4579397

[B97] Thieffry D. (2007). Dynamical roles of biological regulatory circuits. Brief. Bioinform. 8, 220–225. 10.1093/bib/bbm028 17626067

[B98] Thieffry D. Thomas R. (1995). Dynamical behaviour of biological regulatory networks–II. Immunity control in bacteriophage lambda. Bull. Math. Biol. 57, 277–297. 7703921 10.1007/BF02460619

[B99] Thomas R. (1973). Boolean formalization of genetic control circuits. J. Theor. Biol. 42, 563–585. 10.1016/0022-5193(73)90247-6 4588055

[B100] Thomas R. (1978). Logical analysis of systems comprising feedback loops. J. Theor. Biol. 73, 631–656. 10.1016/0022-5193(78)90127-3 703339

[B101] Thomas R. D'Ari R. (1990). Biological Feedback. Boca Raton, FL: CRC Press.

[B102] Thomas R. Gathoye A. M. Lambert L. (1976). A complex control circuit regulation of immunity in temperate bacteriophages. Eur. J. Biochem. 71, 211–227. 10.1111/j.1432-1033.1976.tb11108.x 1009948

[B103] Todd R. G. Helikar T. (2012). Ergodic sets as cell phenotype of budding yeast cell cycle. PLoS ONE 7:e45780. 10.1371/journal.pone.0045780 23049686 PMC3462196

[B104] Traynard P. Fauré A. Fages F. Thieffry D. (2015). Logical modeling of the mammalian cell cycle. viXra:1512.0337. 10.1093/bioinformatics/btw457 27587700

[B105] Tyson J. J. Novák B. (2015). Models in biology: lessons from modeling regulation of the eukaryotic cell cycle. BMC Biol. 13:46. 10.1186/s12915-015-0158-9 26129844 PMC4486427

[B106] Videla S. Guziolowski C. Eduati F. Thiele S. Gebser M. Nicolas J. (2015). Learning Boolean logic models of signaling networks with ASP. Theor. Comp. Sci. 599, 79–101. 10.1016/j.tcs.2014.06.022

[B107] Waltemath D. Adams R. Bergmann F. T. Hucka M. Kolpakov F. Miller A. K.. (2011). Reproducible computational biology experiments with SED-ML–the simulation experiment description markup language. BMC Syst. Biol. 5:198. 10.1186/1752-0509-5-198 22172142 PMC3292844

[B108] Wittmann D. M. Krumsiek J. Saez-Rodriguez J. Lauffenburger D. A. Klamt S. Theis F. J. (2009). Transforming Boolean models to continuous models: methodology and application to t-cell receptor signaling. BMC Syst. Biol. 3:98. 10.1186/1752-0509-3-98 19785753 PMC2764636

[B109] Zañudo J. G. T. Albert R. (2013). An effective network reduction approach to find the dynamical repertoire of discrete dynamic networks. Chaos 23:025111. 10.1063/1.4809777 23822509

[B110] Zhang R. Shah M. V. Yang J. Nyland S. B. Liu X. Yun J. K.. (2008). Network model of survival signaling in large granular lymphocyte leukemia. Proc. Natl. Acad. Sci. U.S.A. 105, 16308–16313. 10.1073/pnas.0806447105 18852469 PMC2571012

