# Supplementary material for: Logical Modeling and Dynamical Analysis of Cellular Networks
Source: Front Genet. 2016 May 31;7:94. doi: 10.3389/fgene.2016.00094 (PMC4885885; doi:10.3389/fgene.2016.00094)
Supplement: Supplementary Figure S1 — This figure provides an illustration of a simple multi-valued model and its asynchronous dynamics represented as a State Transition Graph and as a Hierarchical Transition Graph. [file Image1.pdf]

Supplementary Material: Logical modeling and dynamical analysis of cellular networks

Wassim Abou-Jaoudé, Pauline Traynard, Pedro T. Monteiro, Julio Saez-Rodriguez , Tomáš Helikar, Denis Thieffry, Claudine Chaouiya*

*Correspondence:
Claudine Chaouiya:
chaouiya@igc.gulbenkian.pt

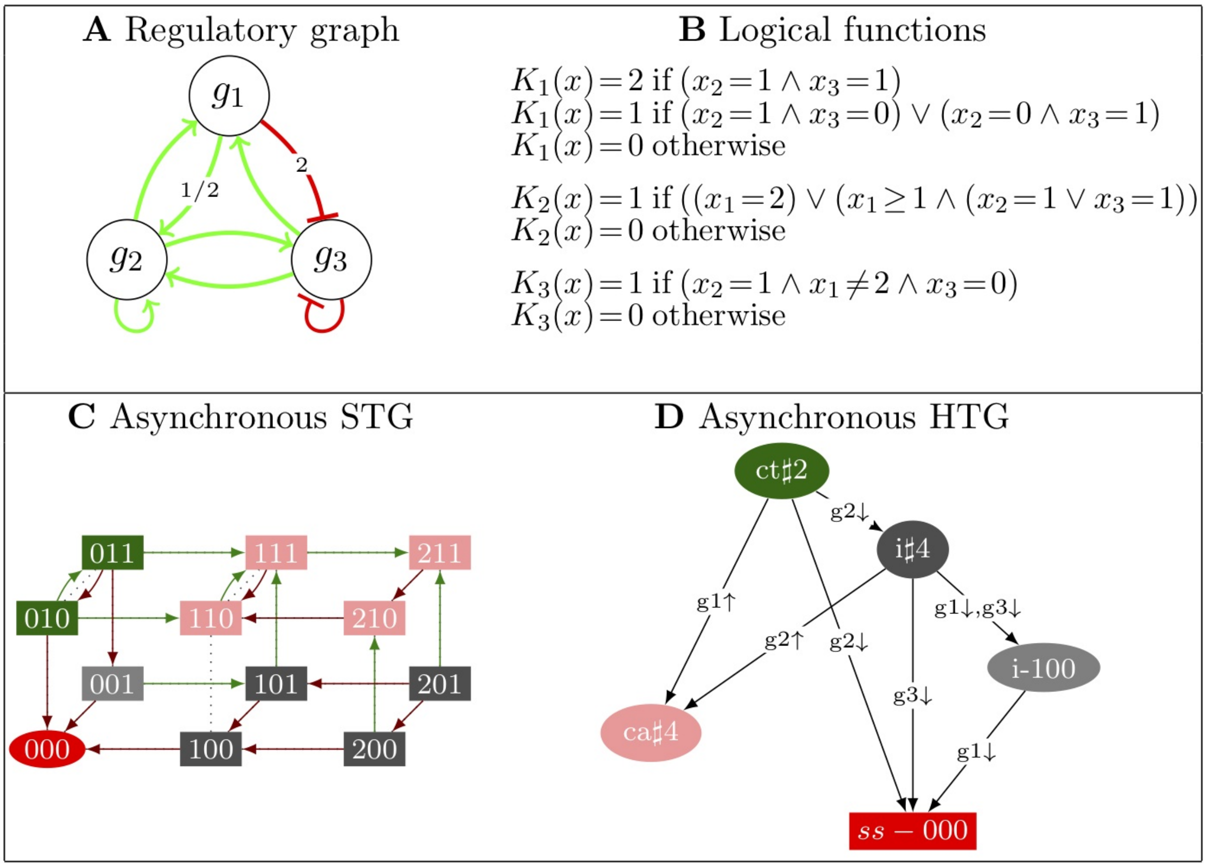

Supplementary Figure S1. Illustration of the basics of the logical formalism - Multi-valued model. (A) The regulatory graph defines the topology of the regulatory structure where nodes represent regulatory components and edges represent regulatory effects (activations denoted in green, inhibitions in red). (B) The logical functions define the values of the variables associated to the regulatory components. Here component g_1 is multi-valued, as opposed to g_2 and g_3, which are Boolean. Note that g_1 inhibitory effect on g_3 only occurs when $x_1 = 2$, i.e. g_1 is at its maximum level; moreover, when at its level 2, g_1 alone is able to activate g_2, but the presence of g_2 or g_3 is needed for this activation to occur when g_1 is at its intermediate level. (C) The asynchronous State Transition Graph (STG), with concurrent transitions from a state, when several variables are called to update their values. The red node is a stable state, the set of light red states is a complex attractor, whereas the cycle in green is transient. (D) The asynchronous Hierarchical Transition Graph (HTG) groups together states that are in the same Strongly Connected Components (SCCs) or from which the same SCCs and/or attractors are reachable (see main text). Each HTG node encompasses the states of the same color in the STG of panel B. Labels on the edges indicate the components updates associated to all the transitions leading from one set of states to another.
